# Identification of a small molecule SR9009 that activates NRF2 to counteract cellular senescence

**DOI:** 10.1111/acel.13483

**Published:** 2021-09-29

**Authors:** Li‐Bin Gao, Ya‐Hong Wang, Zhi‐Hua Liu, Yu Sun, Peng Cai, Qing Jing

**Affiliations:** ^1^ CAS Key Laboratory of Tissue Microenvironment and Tumor Innovation Center for Intervention of Chronic Disease and Promotion of Health Shanghai Institute of Nutrition and Health University of Chinese Academy of Sciences Chinese Academy of Sciences Shanghai China; ^2^ Key Laboratory of Urban Environment and Health Institute of Urban Environment Chinese Academy of Sciences Xiamen China; ^3^ Xiamen Key Laboratory of Physical Environment Xiamen China

**Keywords:** cellular senescence, DNA damage, NRF2, ROS, SASP, SR9009

## Abstract

The senescence‐associated secretory phenotype (SASP) is a striking characteristic of senescence. Accumulation of SASP factors causes a pro‐inflammatory response linked to chronic disease. Suppressing senescence and SASP represents a strategy to prevent or control senescence‐associated diseases. Here, we identified a small molecule SR9009 as a potent SASP suppressor in therapy‐induced senescence (TIS) and oncogene‐induced senescence (OIS). The mechanism studies revealed that SR9009 inhibits the SASP and full DNA damage response (DDR) activation through the activation of the NRF2 pathway, thereby decreasing the ROS level by regulating the expression of antioxidant enzymes. We further identified that SR9009 effectively prevents cellular senescence and suppresses the SASP in the livers of both radiation‐induced and oncogene‐induced senescence mouse models, leading to alleviation of immune cell infiltration. Taken together, our findings suggested that SR9009 prevents cellular senescence via the NRF2 pathway *in vitro* and *in vivo*, and activation of NRF2 may be a novel therapeutic strategy for preventing cellular senescence.

## Graphical abstract

The small molecule SR9009 counteracts cellular senescence induced by persistent DNA damage. The suppressive effects are mediated by inhibiting ROS level through the NRF2 pathway.

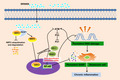

**Abbreviations**
ATM: ataxia telangiectasia‐mutated gene
DDR: DNA damage response
DOX: doxorubicin
DSB: double‐strand break
HDF: human dermal fibroblast
HO‐1: heme oxygenase 1
NQO1: NAD(P)H quinone dehydrogenase 1
NRF2: nuclear factor erythroid‐2‐related factor 2
ROS: reactive oxygen species
SASP: senescence‐associated secretory phenotype
SR: SR9009

## 1 INTRODUCTION

Cellular senescence is a program defined by a perpetual growth arrest of aged or damaged cells (Campisi, 2013; Kuilman et al., 2010). Senescent cells display significant phenotypic changes and alterations in chromatin organization, metabolism, and transcriptional activity. According to the trigger *per se*, senescence can be classified as replicative senescence, oncogene‐induced senescence, and therapy‐induced senescence (Dou et al., 2017; Takahashi et al., 2018). Senescent cells also exhibit increased lysosomal β‐galactosidase activity that is responsible for the characteristic senescence‐associated β‐gal (SA‐β‐gal) staining near neutral pH and secret inflammatory factors with potent effects, including various chemokines, cytokines, extracellular matrix proteins, and growth factors, collectively referred to as the senescence‐associated secretory phenotype (SASP) (Coppe et al., 2008, 2010; Tchkonia et al., 2013). Senescent cells elicit multiple paracrine effects associated with wound healing (Demaria et al., 2014), tissue repair (Demaria et al., 2014; Rhinn et al., 2019), inflammation (Kang et al., 2015) or *in vivo* reprogramming (Mosteiro et al., 2016) through the SASP. Moreover, it can also disrupt tissue homeostasis and promote aging and other pathophysiological diseases, such as fibrosis and cardiovascular disease (Childs et al., 2016; Munoz‐Espin & Serrano, 2014). The SASP reinforces the cell cycle arrest by activating the cell cycle inhibitors p53 and p21^CIP1^ (Acosta et al., 2008, 2013). The activation of the SASP is primarily regulated by the transcription factors nuclear factor κB (NF‐κB), CCAAT/enhancer binding protein β (C/EBPβ), p38 MAPK and GATA4 (Acosta et al., 2008; Chien et al., 2011; Freund et al., 2011; Kang et al., 2015; Kuilman et al., 2008). Therefore, blocking these pathways can be an effective strategy for reducing SASP factors and treatment of senescence‐associated diseases.

Persistent DNA damage can induce cellular senescence. ATM is activated during DNA damage response (DDR) and it phosphorylates the histone variant H2AX at serine 139 (γH2AX). Then, the secondary DDR factors are recruited to the DSB sites to form the DDR foci, including the auto‐phosphorylated form of ATM (p‐ATM), phosphorylated KRAB‐associated protein 1 (pKap1), and p53‐binding protein 1 (53BP1) (Reimann et al., 2010; Xue et al., 2007). Then, p53 is activated and p21 is induced by p53, leading to G1/S cell cycle arrest. Eventually, the cells enter a state of senescence (Ou & Schumacher, 2018). ROS are mediators of DNA damage and an elevated level of reactive oxygen species (ROS) also activates the ATR‐Chk1 axis, leading to the cell cycle arrest (Meng et al., 2018; von Zglinicki, 2002). Alternatively, ROS accumulation can activate the p38/MAPK pathway, which in turn activates and phosphorylates some RNA‐binding proteins responsible for the stabilization of SASP‐dependent mRNA (Tiedje et al., 2012). Consequently, reducing the ROS level may suppress DNA damage and the expression of SASP factors.

Nuclear factor erythroid 2‐related factor 2 (NRF2) is a transcription factor that robustly regulates a series of cytoprotective genes, thereby neutralizing ROS and restoring cellular redox balance (Ge et al., 2017). Under quiescent conditions, KEAP1 represses NRF2 activity, whereas NRF2 is liberated from KEAP1‐mediated repression on exposure to stress, leading to the accumulation and nuclear translocation of NRF2 (Yamamoto et al., 2018). Consequently, NRF2 can bind to the conserved antioxidant response element (ARE) of a series of antioxidative targets, such as NADPH quinone oxidoreductase 1 (NQO1), heme oxygenase‐1 (HO‐1), and then elicits a potent antioxidant response (Jadeja et al., 2020; Kleszczyński et al., 2016). The level of Nrf2 decreases with age; silencing of the Nrf2 gene is associated with the induction of premature senescence and NRF2 deficiency exacerbates cellular senescence promoting chronic inflammation (Fulop et al., 2018; Hiebert et al., 2018; Yuan et al., 2021). Rapamycin can suppress hydrogen peroxide induced premature senescence by activating NRF2 to regulate cell cycle progression (Wang et al., 2017). Kubben et al. reported that the suppressed NRF2 activity and increased oxidative stress account for the Hutchinson‐Gilford progeria syndrome (HGPS), and reactivation of NRF2 reverses premature aging phenotype in HGPS‐induced pluripotent stem cells (iPSCs)‐derived MSCs (Kubben et al., 2016). This suggests that activation of NRF2 may suppress senescence phenotypes.

NR1D1/NR1D2, also called REV‐ERBs, are essential components of the circadian clock, which is involved in tumorigenesis (Sulli et al., 2018), metabolism (Bugge et al., 2012), and inflammation (Griffin et al., 2019). SR9009, a specific agonist of NR1D1/NR1D2, can reduce the weight of obese mice by regulating circadian clock and metabolism (Solt et al., 2012). Also, SR9009 can increase skeletal muscle oxidative capacity and alleviate inflammation in mice (Woldt et al., 2013b). A low concentration of SR9009 enhanced neurite outgrowth during neurogenesis, whereas a high concentration of SR9009 suppressed neurite outgrowth (Shimozaki, 2021). Furthermore, SR9009 had effects on cell proliferation and metabolism independent of REV‐ERBs (Dierickx et al., 2019). However, whether SR9009 can prevent cellular senescence remains unknown. In this study, we identified SR9009 as a promising mitigator that suppresses DNA damage and the SASP by reducing ROS level through activation of the NRF2 pathway.

## 2 RESULTS

### 2.1 SR9009 alleviates the senescence‐associated phenotypes induced by diverse treatments

**FIGURE 1:**
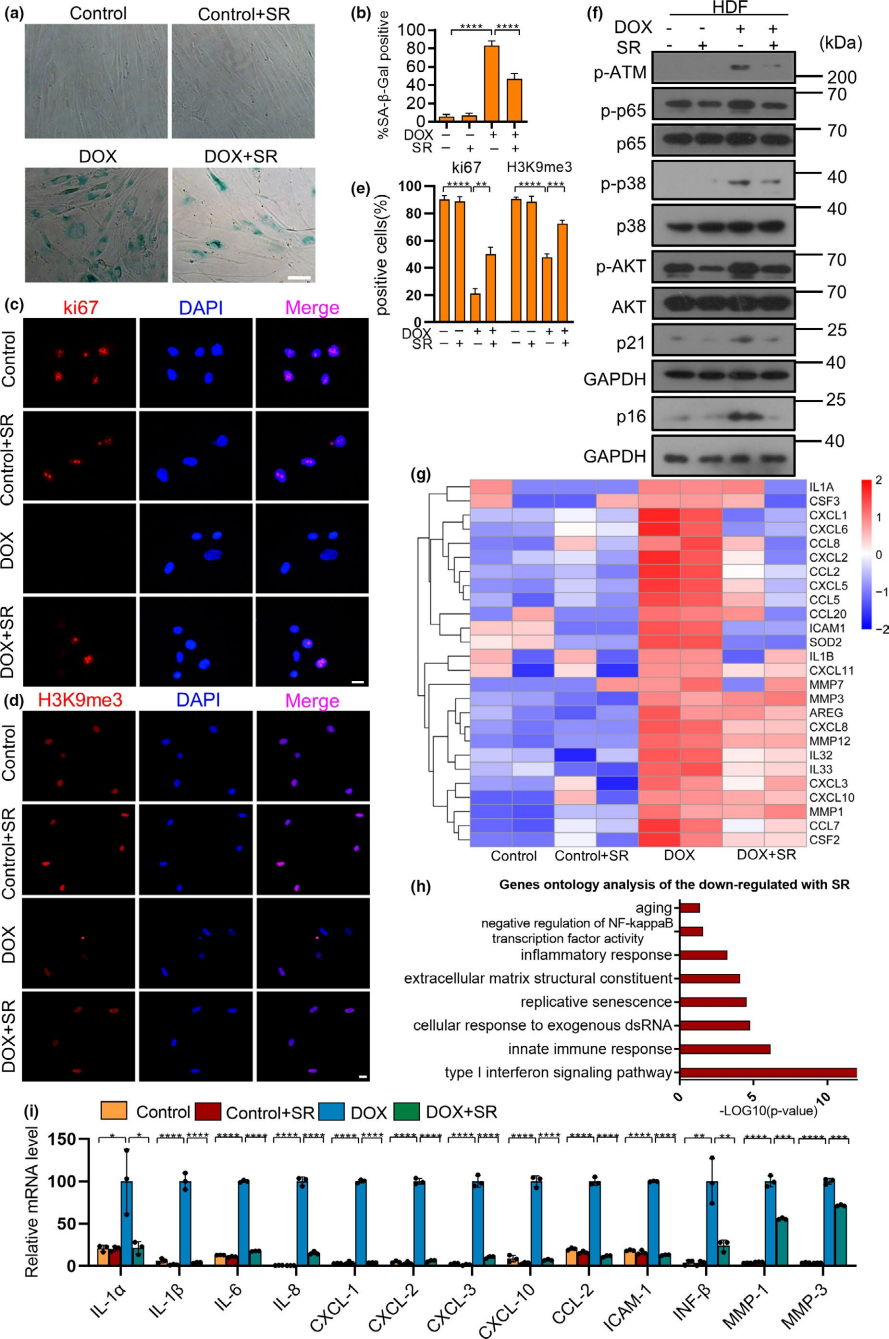
SR9009 attenuates chemotherapy‐induced cellular senescence. (a) Senescence‐associated β‐galactosidase (SA‐β‐Gal) of Control, Control + SR9009, doxorubicin (DOX), DOX + SR9009 HDFs. Scale bar, 50 μm. (b) Quantitative analysis of SA‐β‐Gal positive cells of HDFs in different groups. (c) Immunofluorescence staining for marker of DNA damage (γ‐H2AX [red], and 40,6‐diamidino‐2‐phenylindole [blue]). Scale bar, 20 μm. (d) Immunofluorescence staining for heterochromatin marker (H3K9me3[red], and 40,6‐diamidino‐2 phenylindole [blue]). Scale bar, 20 μm. (e) Quantitative analysis of Immunofluorescence staining results. (f) Western blot analysis of DNA damage factor p‐ATM, p‐p53, cell arrest factor p21 and p16, transcription factor p‐p38, p‐p65 regulating the expression of SASP in different groups. GAPDH was used as loading control. The representative data from three independent experiments are shown. (g) Heat map of SASP factors expression obtained from the transcriptome analysis in doxorubicin‐induced senescence cells following of SR9009 treatment. (h) Top downregulated terms identified after the SR9009 treatment using DAVID gene ontology analysis. (i) RT‐qPCR analysis of SASP factors gene expression, RPL13A was used as loading control. For all graphs, error bars indicate mean ± SEM of triplicate measurements. *p < 0.05, **p < 0.01, ***p < 0.001, ****p < 0.0001; one‐way ANOVA

Human dermal fibroblast cells (HDFs), exhibiting canonical senescence‐associated phenotype, are a typical choice for studying cellular senescence. In order to determine the optimal concentration of SR9009 treatment for HDFs, the cytotoxicity at various concentrations was assessed. Up to 10 μM SR9009 did not cause cell apoptosis of HDFs within 72 h. Thus, we chose the optimal concentration of 10 μM for treatment of HDFs (data not shown). To explore if SR9009 could suppress cellular senescence, HDFs were pretreated with SR9009, followed by adding doxorubicin (DOX) to induce senescence, which represented the universal DNA damage‐induced senescence model. We found that SR9009 decreased the activity of senescence‐associated β‐galactosidase (SA‐β‐Gal) of HDFs (Figure 1a,b, SR denotes SR9009 in the following Figures). Quantification of the proliferation rate by ki67 staining confirmed that SR9009 increased the replicative activity of HDFs (Figure 1c,e), as supported by decreased levels of p21 and p16, key regulators of the cell cycle arrest in eukaryotic cells (Figure 1f). The heterochromatin marker H3K9me3, reduced in senescent cells (Ocampo et al., 2016), was elevated by SR9009 treatment (Figure 1d,e). To systematically explore the altered phenotype, we subsequently performed gene expression profiling with RNA sequencing in SR9009‐treated cells undergoing DNA damage‐induced senescence, using two independent biological replicates. The differentially expressed genes were displayed in the volcano plot (Figure S1A). The RNA sequencing data confirmed that the elevated SASP factors were reduced by SR9009, as shown in the heat map (Figure 1g), including IL‐1α, IL‐1β, IL‐8, IL‐6, and CXCL‐1. Detection of the expression of SASP factors by RT‐PCR also confirmed the result (Figure 1i). The ELISA assay also confirmed that SR9009 reduced content of IL‐1α and IL‐1β in the cultured medium of HDFs (Figure S1G). Gene Ontology (GO) analysis of the top downregulated genes in SR9009‐treated cells revealed significant enrichment of aging, inflammatory response and negative regulation of NF‐κB factor activity (Figure 1h). Gene Set Enrichment Analysis (GSEA) showed the weakened inflammatory response genes, G2M Checkpoint, NF‐κB and PI3K‐AKT pathways (Figure S1B–F). MTT and cell cycle analysis showed increased proliferation capacity and suppressed cell cycle arrest (Figure S1H–I). These results were in accordance with the anti‐senescence role of SR9009.

Then we examined the DNA damage response and NF‐κB pathway that are involved in SASP (Chien et al., 2011; Rodier et al., 2009). SR9009 attenuated the expression of p‐ATM S1981, p‐p53 S15 and compromised the activation of NF‐κB, as measured by phosphorylation of the NF‐κB p65/RelA subunit (Figure 1f). The p38 signaling pathway, which is associated with the regulation of SASP (Alspach et al., 2014), was also attenuated by SR9009 (Figure 1f). A similar response can be observed in different senescence stimuli, including IR‐induced senescence (Kang et al., 2015) (Figure S2), oncogene‐induced senescence (Hari et al., 2019) (Figures S3 and S4) and replicative senescence (Figure S5A). Post‐treatment of SR9009 also had a similar response on HDFs (Figure S5B). SR9011, an SR9009 analogue, could also delay the senescence of HDFs (Figure S5C–D). A similar phenotype was observed in other types of normal human cells (strain IMR‐90 from fetal lung), including chemotherapy‐induced senescence (Figure S6) and oncogene‐induced senescence (Figures S7 and S8). Taken together, these results suggest that SR9009 could suppress the senescence and SASP induced by diverse triggers of cellular senescence.

### 2.2 SR9009 is more effective than rapamycin in reducing the SASP

**FIGURE 2:**
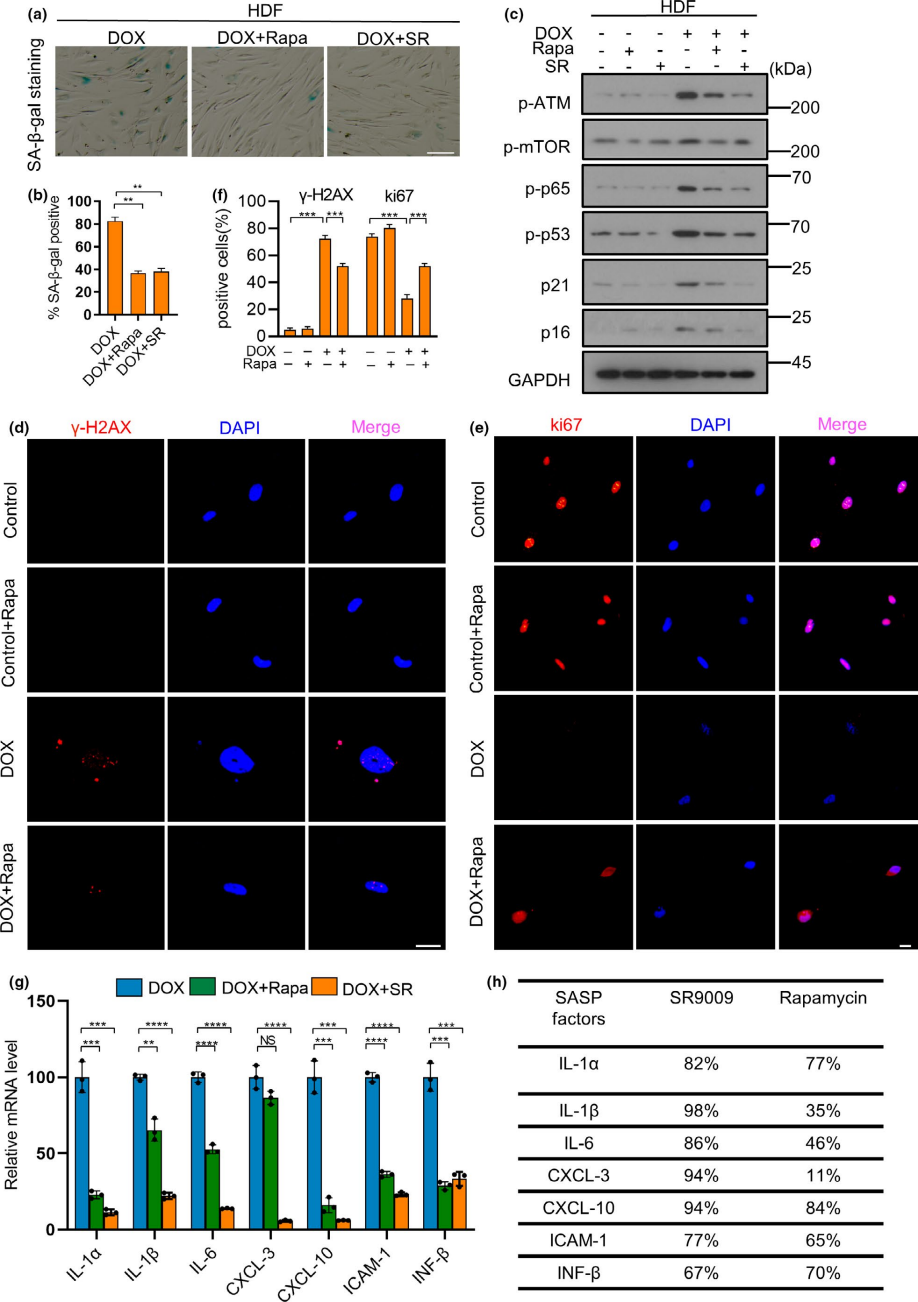
SR9009 is more effective than Rapamycin in reducing the SASP of senescent cells. (a) Senescence‐associated β‐galactosidase (SA‐β‐Gal) of HDFs undergoing chemotherapy‐induced senescence in the absence or presence of Rapamycin and SR9009. Scale bar, 100 μm. (b) Quantitative analysis of SA‐β‐Gal positive cells of HDFs. (c) Western blot analysis of DNA damage factor p‐ATM, p‐MTOR, p‐p53, cell arrest factor p21, transcription factor p‐p65 regulating the expression of SASP. GAPDH was used as loading control. (d) Immunofluorescence staining for marker of DNA damage (γ‐H2AX [red], and 40,6‐diamidino‐2‐phenylindole [blue]). (e) Immunofluorescence staining for proliferation marker (ki67 [red], and 40,6‐diamidino‐2‐phenylindole [blue]). Scale bar, 20 μm. (f) Quantitative analysis of Immunofluorescence staining results. (g) RT‐qPCR analysis of SASP factor gene expression, RPL13A was used as loading control. (h) The table showed the efficiency of SR9009 and Rapamycin suppressing the expression of SASP factors. The representative data from three independent experiments are shown. For all graphs, error bars indicate mean ± SEM of triplicate measurements. *p < 0.05, **p < 0.01, ***p < 0.001, ****p < 0.0001; one‐way ANOVA

Rapamycin, a natural mTOR inhibitor, prevented the senescence and SASP of cells undergoing senescence (Herranz et al., 2015). To verify and compare the effect of rapamycin with SR9009, we pretreated the cells with rapamycin in the optimum concentration (10 nM), which decreased the expression of SASP factors most effective and did not cause apoptosis of HDF cells (data not shown). We found that rapamycin decreased the SA‐β‐Gal activity, increased the proliferation marker ki67 ratio and diminished the expression of most SASP factors of HDFs (Figure 2a–b, e–g). The DNA damage factors p‐ATM, p‐p53 and DNA damage foci were reduced by rapamycin (Figure 2d,f). Surprisingly, SR9009 remarkably reduced the expression of DNA damage factors, p‐p65, p21 and p16 (Figure 2c). Since SASP plays a major pro‐inflammatory role on tissue microenvironment, we then compare the reducing effect of SASP between SR9009 and rapamycin. The results demonstrated that the efficiency of reducing the SASP (i.e., ILs and CXCLs) by SR9009 was higher than by rapamycin (Figure 2g,h). Together, these results suggest that SR9009 is more effective than rapamycin in reducing the SASP, at least partially.

### 2.3 SR9009 is independent of NR1D1 and NR1D2 in preventing the senescence‐associated phenotype

**FIGURE 3:**
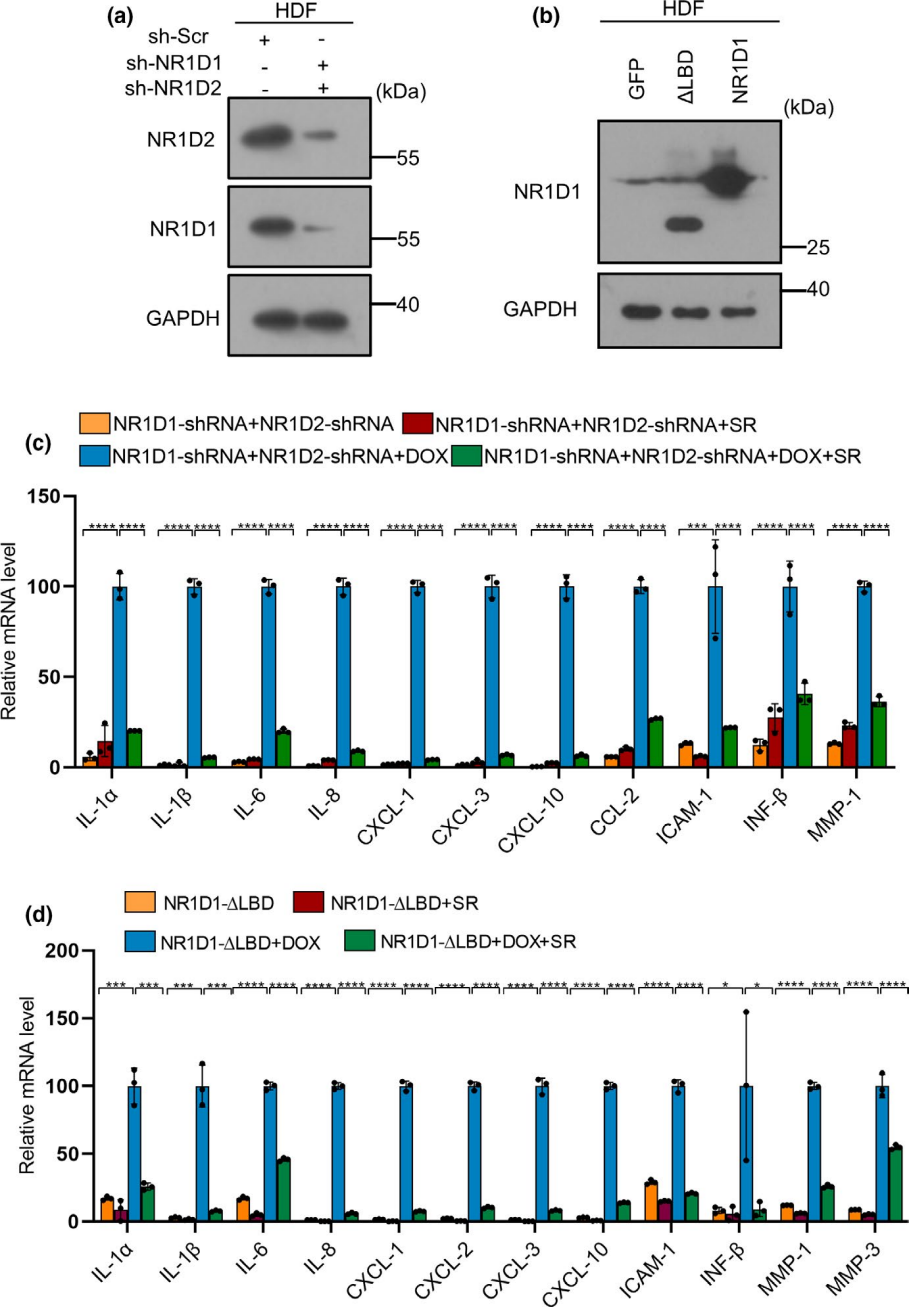
SR9009 alleviates the senescence response independently of NR1D1 and NR1D2. (a) Immunoblot detection of NR1D1 and NR1D2 upon knock down by shRNA. GAPDH was used as loading control. (b) Immunoblot detection of NR1D1‐ΔLBD upon overexpression of NR1D1‐ΔLBD, overexpression of NR1D1 was used as positive control, GAPDH was used as loading control. (c) RT‐qPCR analysis of SASP factor gene expression after SR9009 treatment based on the knock down of NR1D1 of NR1D2. RPL13A was used as loading control. (d) RT‐qPCR analysis of SASP factor gene expression after SR9009 treatment based on overexpression of NR1D1‐ΔLBD. RPL13A was used as loading control. The representative data from three independent experiments are shown. For all graphs, error bars indicate mean ± SEM of triplicate measurements. *p < 0.05, **p < 0.01, ***p < 0.001, ****p < 0.0001; one‐way ANOVA

SR9009 was previously reported as a synthetic NR1D1 and NR1D2 ligand (Solt et al., 2012). We tested whether increasing NR1D1 expression could prevent senescence phenotypes. As shown in Figure S9A, B, overexpressed NR1D1 did not decrease the SA‐β‐Gal of HDFs. On the contrary, it increased the expression of SASP factors and aggravated the DNA damage response (Figure S9C, D). This result was consistent with a previous report that SR9009 had NR1D1‐independent effects (Dierickx et al., 2019). We further validated that neither overexpressing NR1D1‐ΔLBD lacking the ligand domain (Ka et al., 2017) nor knocking down NR1D1 and NR1D2 simultaneously by shRNA (Figure 3a,b) abolished the SR9009’s effects on suppressing the expression of SASP factors (Figure 3c,d). These results suggested that NR1D1 and NR1D2 were not involved in SR9009‐mediated delay of senescence.

### 2.4 Decreased ROS level accounts for the anti‐senescence effect of SR9009

**FIGURE 4:**
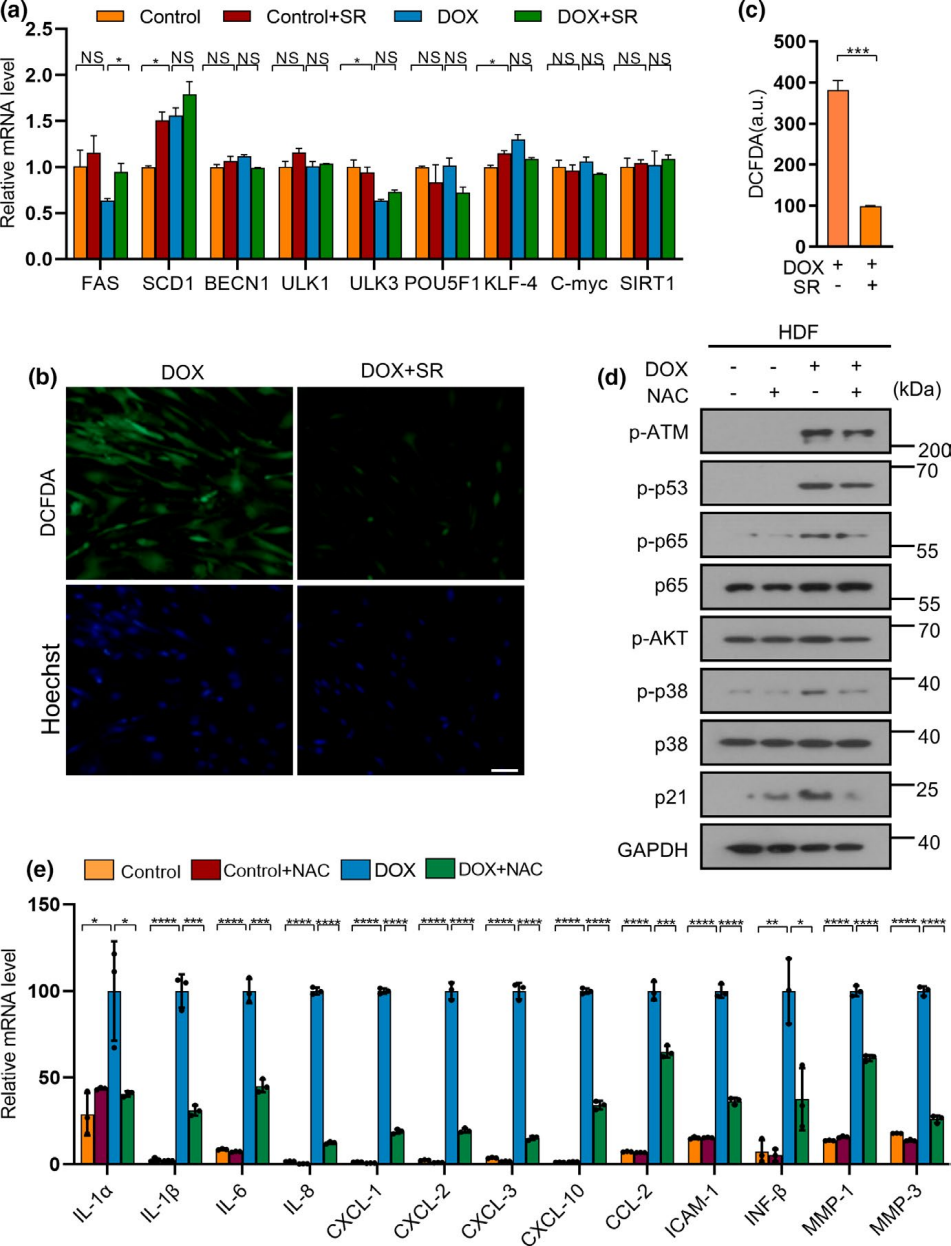
SR9009 decreases the ROS level of HDF cells undergoing chemotherapy‐induced senescence response. (a) RT‐qPCR detection of the expression of lipogenic enzymes, core autophagy genes and pluripotency‐related genes in HDFs undergoing chemotherapy‐induced senescence after SR9009 treatment. RPL13A was used as loading control. (b) Analysis of reactive oxygen species (ROS) level by H2DCF‐DA staining in HDFs pretreated or not (control) with SR9009. Scale bar, 50 μm. (c) Quantitative analysis of the fluorescence intensity stained by H2DCF‐DA. (d) Western blot analysis of DNA damage factor p‐ATM, p‐p53, cell arrest factor p21, transcription factor p‐p38, p‐p65 regulating the expression of SASP. GAPDH was used as loading control. (e) RT‐qPCR analysis of SASP factor gene expression of HDFs after NAC treatment. RPL13A was used as loading control. The representative data from three independent experiments are shown. For all graphs, error bars indicate mean ± SEM of triplicate measurements. *p < 0.05, **p < 0.01, ***p < 0.001, ****p < 0.0001; Student's *t*‐test (c) and one‐way ANOVA for all others

Previous study showed SR9009 could induce the apoptosis of cancer cells by suppressing autophagy and *de novo* lipogenesis through repressing fatty acid synthase (FAS) and stearoyl‐CoA desaturase 1 (SCD1) (Sulli et al., 2018). We thus detected the expression of lipogenic enzymes and core autophagy genes. SR9009 slightly increased the expression of FAS and did not alter the expression of SCD1 and core autophagy genes (Figure 4a). Cellular senescence‐related SIRT1 and several pluripotency‐related genes, whose higher expression can reverse the senescence state (Ocampo et al., 2016), were also examined and showed no significant difference (Figure 4a). A slightly increased but not significant difference change was observed in HDFs undergoing oncogene‐induced senescence (Figure S10A). We then detected the ROS level in HDFs after the SR9009 treatment. The ROS level and mitochondrial ROS production were significantly reduced by SR9009 (Figure 4b,c, Figure S10B, C and Figure S11A, B). Meanwhile, SR9009 prevented the decrease of JC‐1 aggregate/JC‐1 monomer ratio (indicator for mitochondrial membrane potential) (Figure S11C, D). This suggests that SR9009 was linked to mitochondrial activity. We then tested whether the decreased ROS accounted for alleviating senescence and SASP. The data showed that NAC, an ROS inhibitor, could alleviate senescence, SASP and DNA damage response in chemotherapy‐induced senescence (Figure 4d,e), as well as in oncogene‐induced senescence (Figure S12). Taken together, these results suggest that the reduced ROS could suppress senescence and SASP.

### 2.5 The anti‐senescence effect of SR9009 is largely dependent on the NRF2 pathway

**FIGURE 5:**
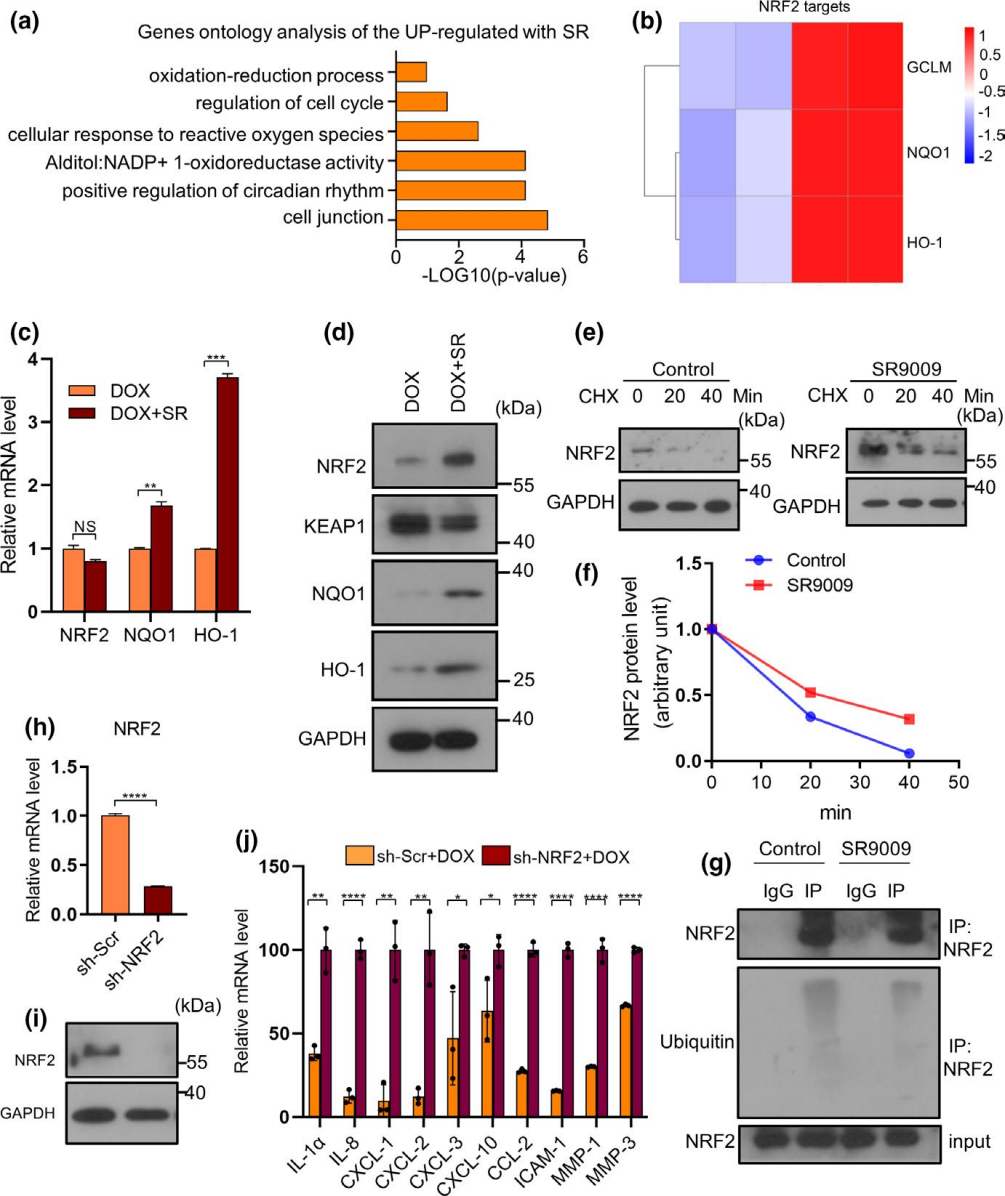
SR9009 increased the expression of NRF2 by preventing proteasome‐mediated NRF2 degradation. (a) GO analysis of upregulated genes after treatment by SR9009 in HDFs undergoing doxorubicin‐induced senescence. (b) NRF2 targets shown by Heatmap in our RNA‐ sequencing data. (c) RT‐qPCR analysis of NRF2 and NRF2 targets. RPL13A was used as loading control. (d) Western blot analysis of NRF2 and NRF2 targets after SR9009 treatment. GAPDH was used as loading control. (e) Western blot analysis of NRF2 level in the control and SR9009‐treated cells in the presence of 100 mg/ml CHX for the indicate time periods. (f) Quantification of the NRF2 band intensity by ImageJ in the control and SR9009‐treated HDFs in the presence of 100 mg/ml CHX for the indicate time periods. NRF2 levels in the untreated cells were normalized to 1. (g) Ubiqutin‐Nrf2 was determined by immunoprecipitation (IP) of Nrf2 with a subsequent WB assay with anti‐ubiquitin antibody in control and SR9009 treated cells. (h) RT‐qPCR analysis of NRF2 expression after knockdown by shRNA. RPL13A was used as loading control. (i) Western blot detection of the expression of NRF2 after knockdown by shRNA. (j) RT‐qPCR analysis of SASP factor gene expression of HDFs harboring NRF2 knock down undergoing chemotherapy‐induced senescence. RPL13A was used as loading control. The representative data from three independent experiments are shown. For all graphs, error bars indicate mean ± SEM of triplicate measurements. *p < 0.05, **p < 0.01, ***p < 0.001, ****p < 0.0001; Student's *t*‐test (f, g) and one‐way ANOVA for all others

**FIGURE 6:**
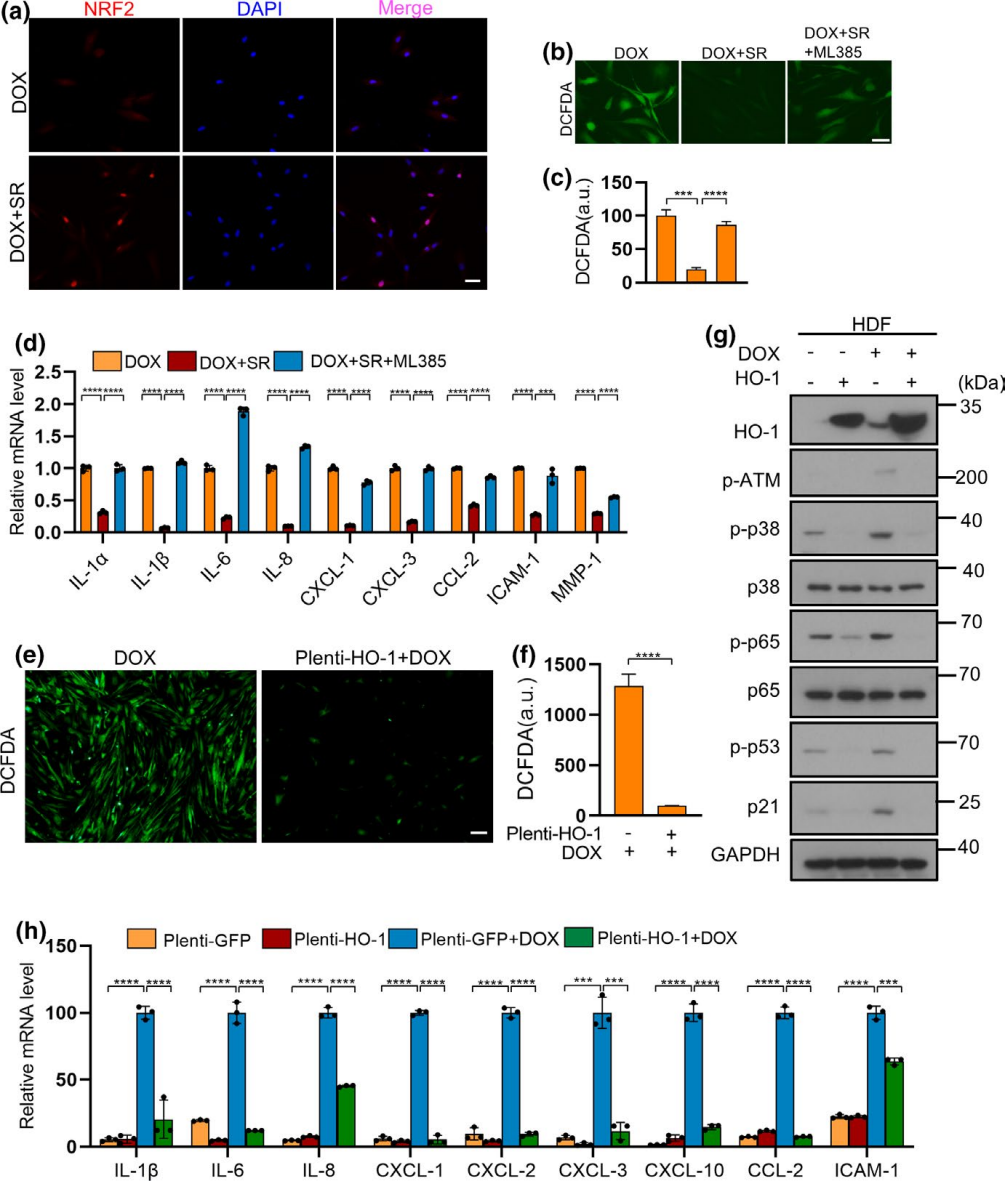
SR9009 suppresses senescence phenotypes of HDFs by activating NRF2 pathway. (a) Immunofluorescence staining of NRF2 after treatment by SR9009 in HDFs undergoing doxorubicin‐induced senescence. Scale bar, 50 μm. (b) Analysis of reactive oxygen species (ROS) level by H2DCF‐DA staining in HDFs pretreated or not (control) with SR9009 and ML385. Scale bar, 10 μm. (c) Quantitative analysis of the fluorescence intensity stained by H2DCF‐DA. (d) RT‐qPCR analysis of SASP factor gene expression of HDFs after ML385 treatment undergoing chemotherapy‐induced senescence. RPL13A was used as loading control. (e) DCFDA‐based ROS quantification in HDF cells overexpressing HO‐1 or control cells undergoing doxorubicin‐induced senescence. Scale bar, 50 μm. (f) Quantitative analysis of reactive oxygen species (ROS) level. (g) Western blot analysis of DNA damage factor p‐ATM, p‐p53, cell arrest factor p21, transcription factor p‐p38, p‐p65 regulating the expression of SASP. GAPDH was used as loading control. (h) RT‐qPCR analysis of SASP factor gene expression of HDFs after overexpression of HO‐1. The representative data from three independent experiments are shown. For all graphs, error bars indicate mean ± SEM of triplicate measurements. *p < 0.05, **p < 0.01, ***p < 0.001, ****p < 0.0001; Student's *t*‐test (e) and one‐way ANOVA for all others

Gene ontology analysis of upregulated genes after SR9009 treatment in chemotherapy‐induced senescence indicated a significant enrichment in cellular response to reactive oxygen species, oxidation‐reduction process, and regulation of the cell cycle (Figure 5a). NRF2 is one of the master regulators of antioxidant responses and a critical redox sensor. It binds to AREs and activates the transcription of various antioxidant genes that are known for counteracting ROS (Gorrini et al., 2013), and it may account for the decreased ROS level. The protein level of NRF2 was markedly increased in HDFs treated with SR9009 (Figure 5d). However, the mRNA expression level of NRF2 was slightly reduced, though not significantly (Figure 5c). This leads us to consider that the changes in NRF2 protein expression level may be due to post‐transcriptional rather than transcriptional regulation. Cycloheximide (CHX) was added for 0, 20 min, or 40 min respectively into SR9009‐treated HDFs cells. The level of NRF2 protein gradually decreased in SR9009‐treated cells after CHX treatment. By analyzing the quantification curve, the half‐life of NRF2 appeared to be around 13 min in control cells and 23 min in SR9009‐treated cells, implying that SR9009 may post‐transcriptionally regulate the protein expression of NRF2 (Figure 5e,f). Ubiquitination and subsequent proteasome‐mediated protein degradation may account for the regulation of the expression level of NRF2 in cells. One speculation is that the higher levels of NRF2 in SR9009‐treated cells may act via preventing proteasome‐mediated NRF2 degradation. Immunoprecipitation assay with NRF2 antibody revealed that a slightly lower level of Ub‐NRF2 was detected in SR9009‐treated cells, suggesting that SR9009 prevents ubiquitination of NRF2 (Figure 5g). To further determine the role of NRF2 in the inhibition of cellular senescence and SASP, knockdown of NRF2 was performed and showed an increase of SASP (Figure 5h–j). Furthermore, ML385, a NRF2 inhibitor (Singh et al., 2016), significantly blocked the suppressive effects of SR9009 in SASP factors and ROS level (Figure 6b–d). These results suggested that NRF2 is required in SR9009 mitigated senescence.

Immunofluorescence analysis showed the nuclear NRF2 expression was increased after SR9009 treatment (Figure 6a). Nuclear NRF2 activates the transcription of a number of antioxidant genes that are known for counteracting ROS, including NQO1 and HO‐1. This was consistent with the RNA sequencing result showing upregulation of NQO1 and HO‐1, as depicted in the heatmap (Figure 5b). The elevated expressions of HO‐1 and NQO1 induced by SR9009 were also verified (Figure 5c,d). Oltipraz, a small molecular compound that can induce NRF2 nuclear translocation, effectively reduces senescence, SASP and DNA damage of HDFs (Figure S13A, D). Oltipraz also had the same effects on HDFs undergoing oncogene‐induced senescence (Figure S13B, E). Another NRF2 activator THBQ also had similar response (Figure S13C, F). These results lead us to explore whether the increased expression of NQO1 and HO‐1, downstream target genes of NRF2, could markedly reduce the ROS level to alleviate the senescence and SASP. We identified that overexpression of HO‐1 and NQO1 alleviates senescence, SASP and DNA damage response (Figure 6e–h and Figure S14). As supporting evidence, we observed a similar response in oncogene‐induced senescence (Figures S15, S16). Considering NRF2 had more target genes in addition to NQO1 and HO‐1, other NRF2 targets may also be involved in the event. Collectively, these results suggest that SR9009 prompts the expression and nuclear translocation of NRF2, thereby regulating target genes expression to reduce ROS level, thus alleviating senescence.

### 2.6 SR9009 alleviates the senescence‐associated phenotypes *in vivo*

**FIGURE 7:**
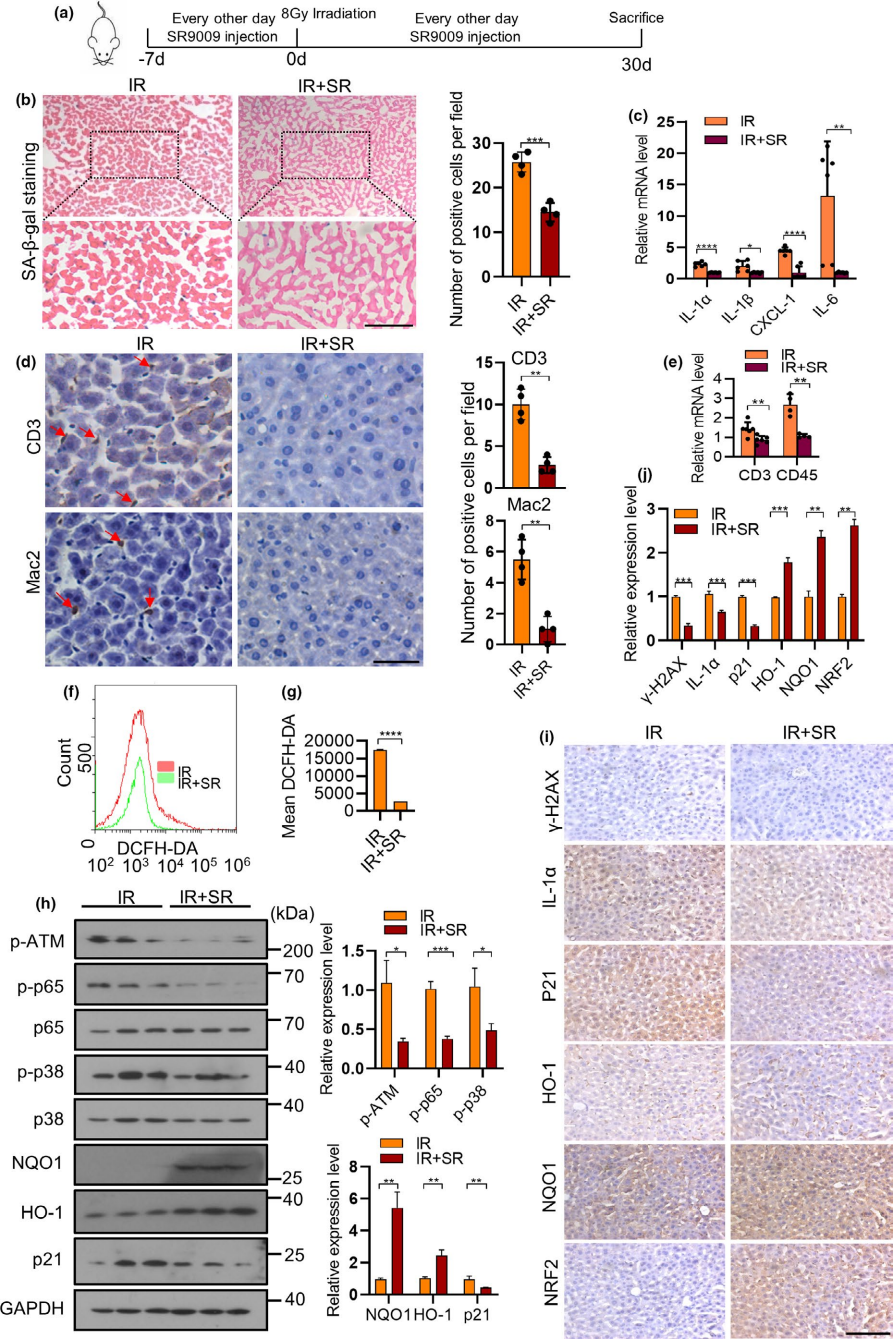
SR9009 prevents the IR‐induced senescence phenotypes *in vivo* via NRF2 pathway. (a) Experimental design of *in vivo* studies. C57BL/6 mice were administered SR9009 every other day, then induced by IR to promote senescence *in vivo*. 30 days after ionizing Irradiation, mice were sacrificed for analysis. (b) Liver tissues were analyzed by SA‐β‐gal staining. Scale bar, 100 μm (left). Quantitative analysis of SA‐β‐Gal positive cells of liver tissues (right). (c) RT‐qPCR analysis of SASP factor gene expression in livers described in (a). (d) Immunohistochemistry staining of CD3, Mac2 in liver tissues of IR and IR + SR9009 group. Scale bar, 50 μm. (left). Quantitative analysis of immunohistochemistry staining (right). (e) RT‐qPCR analysis of CD3, CD45 in mouse livers described in (a). (f) Analysis of reactive oxygen species (ROS) level by flow cytometry in liver tissues of the IR and IR + SR9009 group. (g) Quantitative analysis of reactive oxygen species (ROS) level in liver tissues of the IR and IR + SR9009 group. (h) Immunoblot detection of proteins in livers of the IR and IR + SR9009 group (left). Quantitative analysis of immunoblot results (right). (i) Immunohistochemistry staining of indicated genes in livers of IR and IR + SR9009 group using antibodies shown left. Scale bar, 100 μm. (j) Quantitative analysis of Immunohistochemistry staining (right). The results are representative of three independent experiments. *p < 0.05, **p < 0.01, ***p < 0.001, ****p < 0.0001; Student's *t*‐test

We want to know whether the SR9009 can suppress senescence and SASP *in vivo*. SR9009‐treated mice were exposed to sub‐lethal doses of ionizing irradiation that induced DNA damage, senescence, and the SASP as described previously (Dou et al., 2017; Freund et al., 2012; Kang et al., 2015; Le et al., 2010; Rodier et al., 2009). SR9009 treatment persisted every other day until 30 days post irradiation (Figure 7a). We focused primarily on the liver, because liver was considered as the main organ associated with chronic inflammation leading to multiple diseases, including fatty liver and diabetes (Furman et al., 2019; Papatheodoridi et al., 2020). SR9009 decreased SA‐β‐Gal and SASP factors, including IL‐1α, IL‐1β, IL‐6, CXCL‐1 in liver tissue (Figure 7b,c). SASP factors could alter the tissue microenvironment through recruiting immune cells and modulating their activity, causing chronic inflammation. As expected, SR9009 treatment decreased the accumulation of T cell markers CD3 and macrophage markers Mac2 determined by both immunohistochemical staining (Figure 7d, red arrow indicated) and qPCR analysis (Figure 7e). The DNA damage response factors p‐ATM, γ‐H2AX were markedly reduced by SR9009 (Figure 7h–j). SR9009 decreased the ROS level (Figure 7f,g), upregulated the expression level of NRF2, NQO1, and HO‐1 (Figure 7h–j) of liver. S9009 had no impact in normal tissues examined by DNA damage indicator γ‐H2AX (Figure S17A,B). We also verified that SR9009 decreased the activity of SA‐β‐Gal of other organs, including the lung (Figure S17C,D). These results were in accordance with our *in vitro* results, suggesting SR9009 may alleviate the senescence response via the NRF2 pathway.

Finally, we employed an animal model of oncogene‐induced senescence, wherein oncogenic Nras^G12V^ is stably delivered into mouse livers by hydrodynamic injection of transposable elements (Kang et al., 2011). The results showed that SR9009 treatment reduced the SA‐β‐Gal ratio (Figure S18A) and immune cell infiltration of liver tissues (Figure S18B, red arrow indicated). SR9009 also decreased the DNA damage factors and upregulated the expression of NRF2, NQO1 and HO‐1 (Figure S18B,C). Meanwhile, SR9009 increased the lifespan of *Caenorhabditis elegans* compared with control group and rapamycin treatment group (Figure S19A), as well as reduced the ROS level (Figure S19B,C). Taken together, these results demonstrated that SR9009 can suppress senescence and SASP via the NRF2 pathway *in vivo*.

## 3 DISCUSSION

SR9009, previously reported as a small molecule ligand for NR1D1 and NR1D2, had an important role in feedback regulation of the circadian oscillator and reversing obesity (Solt et al., 2012). It can also increase the exercise capacity of mice by regulating mitochondrial biogenesis and autophagy, and reduce the severity of fulminant hepatitis in mice (Pourcet et al., 2018; Woldt et al., 2013a). However, there were no reports regarding the role of SR9009 in cellular senescence. Our findings demonstrated that SR9009 suppresses the senescence, DNA damage and SASP of cells undergoing senescence induced by different types of stress. Furthermore, the advantage of SR9009 was that it was more effective than rapamycin in reducing the SASP. We next revealed that SR9009 suppressed cellular senescence independent of NR1D1 and NR1D2 and counteracted cellular senescence through reducing ROS level via the NRF2 pathway.

SASP is a key feature of senescent cells and its accumulation can cause long‐term chronic inflammation *in vivo*. DNA damage induces NF‐kB activation, then binding to the promoter of some SASP factors and regulating their expression (Rodier et al., 2009)^,^(Sun et al., 2012; Zhang et al., 2018), suggesting a close relationship between DNA damage and SASP induction. P38 can regulate NF‐kB activity and stabilize SASP effector mRNA stability post‐transcriptionally (Alspach et al., 2014; Freund et al., 2011). The DDR factors, including p‐ATM, p‐p53, and γH2AX were decreased by SR9009. The activation of the NF‐κB pathway and P38 pathway was compromised by SR9009. This implies SR9009 can regulate the upstream molecule of DDR pathway which may affect the expression of SASP. P21, the crucial regulator of senescent cell cycle arrest, was also suppressed by SR9009 and the cell cycle arrest was partially abolished. The decreased expression of p21 may be attributed to the decreased activation of DNA damage factor p53, which regulates the expression of p21. Our results also showed that the level of H3K9me3, a dysregulated heterochromatin marker during aging (Benayoun et al., 2015), was raised by SR9009. This implies SR9009 may act on the upstream molecular which causes DNA damage and epigenetic change during cellular senescence.

Interestingly, our study showed that SR9009 alleviated senescence, DNA damage and SASP independent of NR1D1 and NR1D2. This result was consistent with the previous report that SR9009 had NR1D1‐independent effects on cell proliferation and metabolism (Dierickx et al., 2019). So, we next found that SR9009 suppressed senescence, DNA damage and SASP by decreasing the ROS level. ROS can induce growth arrest *in vitro* by triggering the DNA damage response (DDR) response (Cameron et al., 2019). In contrast, in response to persistent DNA damage, p38‐MAPK‐mediated mitochondrial dysfunction and ROS production can activate p21, inducing cell cycle arrest (Borodkina et al., 2014). Besides, IL‐6, a major SASP factor, can elevate the intracellular level of ROS to induce DNA damage (Wassmann et al., 2004). Therefore, it is conceivable that there was a feedback loop between DNA damage and ROS collectively driving senescence and SASP. Consequently, suppressing the ROS level can reduce DNA damage and SASP to alleviate cellular senescence.

SR9009 increased the expression and nuclear translocation of NRF2. NRF2 binds to the AREs and activates the transcription of a number of antioxidant genes that are known for counteracting ROS to alleviate senescence (Gorrini et al., 2013). This was supported by our results that both treatment of NRF2 activators and overexpression the NRF2’s target genes NQO1 and HO‐1 effectively reduced senescence and DNA damage. The accumulation of NRF2 occurred at the post‐transcriptional level, but not at the transcriptional level. Our results showed that SR9009 inhibited Ubiquitin‐Proteasome degradation of NRF2, leading to the increased expression of NRF2. KEAP1 can induce ubiquitin‐proteasome degradation of NRF2 by serving as a substrate scaffold for Cul3‐containing E3 ubiquitin ligase; the DGR domain of KEAP1 is critical for binding with NRF2 (Ge et al., 2017). We speculated that SR9009 bound directly to the DGR domain of KEAP1, leading to the disruption of the interaction between NRF2 and KEAP1. Consequently, the degradation of NRF2 was blocked, resulting in the nuclear accumulation of NRF2. How SR9009 downregulated the expression of KEAP1 and whether SR9009 binds directly to KEAP1 warrants further investigation.

We found that the SR9009 compromised the senescence response in distinct models of cellular senescence, including chemotherapy‐induced senescence, IR‐induced senescence, and oncogene‐induced senescence. SR9009 also inhibited senescence, DNA damage and SASP in IMR90 fibroblast cells. We further found that SR9009 suppressed senescence, DNA damage and SASP *in vivo* under the IR‐induced senescence model and the oncogene‐induced senescence model. Released SASP into tissue microenvironment can cause chronic inflammation. SR9009 effectively decreased the immune cell infiltration, suggesting that SR9009 can reduce chronic inflammation *in vivo*; may subsequently alleviate chronic inflammation‐associated human diseases, that is, fatty liver, diabetes, atherosclerosis. However, it needs to be verified in other *in vivo* senescence models or chronic disease models, including old mice, progeria mice, diabetes, fatty liver, and atherosclerosis, etc.

In summary, our study identified SR9009 could suppress senescence, DNA damage and SASP *in vitro* and *in vivo* through decreasing the ROS level by activating the NRF2 pathway. Further investigations are warranted to elucidate whether SR9009 can treat chronic inflammation diseases.

## 4 MATERIALS AND METHODS

### 4.1 Cell culture and treatment

Human embryonic kidney (HEK) 293T, primary HDFs (human dermal fibroblast) and IMR90 fibroblasts were obtained from the American Type Culture Collection. The cells were grown in DMEM supplemented with 100 units/ml penicillin (Invitrogen), 100 units/ml streptomycin (Invitrogen), and 10% fetal bovine serum (FBS), and were tested for mycoplasma intermittently. The cells were cultured under 5% CO_2_ at 37°C. For doxorubicin‐induced senescence, HDF cells at approximately 60–70% confluence were treated with 300 nM doxorubicin for 24 h. The media were replaced every 3 days and cells were harvested at day 7 for further analysis. For IMR90 cells, 1 μM doxorubicin was used. For oncogene‐induced senescence, HDFs and IMR90s stably expressing HRas^G12V^ were constructed for further analysis. Chemical inhibitors or agonists used were 10 μM SR9009 (MCE), 10 μM Oltipraz (MCE), 10 mM NAC (MCE), 20 μM ML385 (MCE) and 50 μM cyclohexane (MCE).

### 4.2 Retrovirus and lentivirus

Retroviral vectors used in this study were shRNAs targeting NR1D1, NR1D2, NRF2, subcloned into PLKO.1 vector. The shRNA targeting sequences were as follows: NR1D1‐shRNA, CCAGCCCTGAATCCCTCTATA; NR1D2‐shRNA, ATGGTACGGTTCGCATCATTA, NRF2‐shRNA, CCGGCATTTCACTAAACACAA. For construction of overexpression vectors, human HO‐1, NQO1, NR1D1, NR1D1‐∆LBD PCR fragment were subcloned into plenti‐Puro. For retroviral production, retroviral vectors were co‐transfected with VSVG envelope vector and Dr8.2 helper vector into HEK293T cells using Lipofectamine 2000 Transfection Reagent (Invitrogen). The Viral supernatant was collected from the medium of HEK293T cells 2 days after transfection and passed through a 0.45 μm syringe filter (Merck Millipore) to eliminate cells. HDF fibroblasts or IMR‐90s were infected by exposure to virus‐containing medium for 12 to 24 h, followed by selection in puromycin.

### 4.3 SA‐β‐galactosidase staining

For SA‐β‐gal staining, the cells in six well plates were fixed by β‐galactosidase staining fixation solution for 10 min according to SA‐β‐gal staining kit (Beyotime), then washed by PBS and stained by SA‐β‐gal solution prepared according to the instruction manual at 37°C overnight. Then the cells were viewed by light microscopy and the blue‐stained cells were identified as senescent cells. The bule‐stained cells in 5 random fields were applied to calculate the percentage of SA‐β‐gal positive cells. For SA‐β‐Gal activity in the tissues, 5 μm frozen sections were fixed with β‐galactosidase staining fixation solution for 10 min, washed with PBS and were incubated in SA‐β‐Gal staining solution at 37°C overnight.

### 4.4 Quantitative real‐time PCR

Total RNA was extracted with Trizol (Invitrogen), cDNA synthesis was done following the manufacturer protocol (FastQuant RT Kit, TIANGEN BIOTECH, KR106). Real‐time PCR was performed using a SYBR Green qPCR master mix (Toyobo, Osaka, Japan) according to the manufacturer's protocol. The qPCR reactions were run on a QuantStudio 7 Real‐Time PCR system or a 7900HT Fast qPCR instrument (Thermo Fisher Scientific). The primers for the qRT‐PCR were listed in Table S1. All data were normalized to the control using RPL13A or GAPDH as internal control.

### 4.5 Immunoblots

Total cells or tissues were lysed using ice‐cold RIPA buffer containing Phosphatase Inhibitor Cocktail (MCE) and protease inhibitor cocktail (Roche) at 4°C for 30 min. Total Proteins were acquired from the supernatant by centrifugation at 12,000 g for 30 min. Protein concentrations were determined using a BCA kit (Beyotime) and normalized to the lowest concentration. Equal amounts of protein were electrophoresed in 8–12% Tris‐glycine SDS‐PAGE gel according to the molecular weight. Subsequently, the proteins were transferred to the PVDF membrane (Millipore). Membranes were blocked with 5% nonfat milk and incubated with primary antibodies at 4°C overnight. Anti‐mouse or anti‐rabbit HRP‐conjugated secondary antibodies were incubated for 1 h at room temperature, Proteins were visualized with the enhanced chemiluminescence substrate ECL (Thermo Scientific).

### 4.6 IP Analysis

For IP assays, cells were lysed and washed in IP lysis buffer (Beyotime) supplemented with Phosphatase Inhibitor Cocktail (MCE) and protease inhibitor cocktail (Roche). Cell debris were removed by centrifugation, and lysates were incubated at 4°C overnight with protein A/G agarose beads (Beyotime) and NRF2 antibody. The IP were washed three times in IP buffer, Next, boiled and analyzed by Immunoblots (IB) according to the standard methods.

### 4.7 Immunofluorescence

The cells were plated in six well plates covered by cover slip at a density of 10^6^ cells/well. The next day, the cells were fixed with 4% paraformaldehyde for 10 min, then washed by PBS, permeabilized in 0.1% Triton X‐100 for 10 min and blocked with 5% BSA for 1 h at room temperature. Following this, the cells were incubated with primary antibody at 4°C overnight, then washed by PBS and incubated with the secondary antibody for 1 h at room temperature. Then nuclei were stained with DAPI, then mounted by Dako (fluorescent mounting medium). Images were obtained by an inverted fluorescence microscope (Zeiss A1) or a confocal laser scanning microscope (Zeiss LSM700).

### 4.8 Immunohistochemistry (IHC)

Tissues were fixed with 4% paraformaldehyde overnight, then washed by running water. Finally, tissues were embedded in paraffin according to the standard protocol. 5 μm sections were prepared, deparaffinized by xylene Ⅰ for 10min and xylene Ⅱ for10 min. Subsequently, the sections were dehydrated by gradient alcohol, washed by PBS. Antigen retrieval were done using sodium citrate buffer (YEASEN, 36319ES60), then blocked with 3% goat serum, incubated by primary antibody at 4°C overnight, then done using an UltraSensitive™ SP (Mouse/Rabbit) IHC Kit (MXB, KIT‐9710). Signal detection was applied by DAB systems (MXB, MAX‐001). Nuclear was stained by Mayer's hematoxylin (Beyotime). The sections were then dehydrated by gradient alcohol, transparentized with xylene, then were mounted with neutral resin for light microscopy.

### 4.9 Antibodies

Detailed information about the antibodies used including their dilutions and resources (company names, catalog numbers) were provided in Table S2.

### 4.10 Reactive oxygen species detection

Fluorescent dye 2′,7′‐dichlorofluorescin diacetate (H2DCF‐DA; Beyotime) were used to measure the intracellular ROS according to the manufacturer's instructions. Mitochondrial ROS were examined by MitoSOX (YEASEN) according to the manufacturer's instructions.

### 4.11 Mitochondrial membrane potential measurement

The mitochondrial membrane potential in HDFs was measured by JC‐1 (Beyotime, Shanghai, China) according to the manufacturer's instructions. Briefly, the cells were washed with PBS and then incubated with JC‐1 fluorescent dye (10 μmol/L) for 20 min at 37°C in the dark. Stained cells were washed with JC‐1 staining buffer (1×) and maintained on ice. The images of the stained cells were captured by a laser scanning confocal microscope.

### 4.12 Cell cycle analysis

For cell cycle analysis, cells were collected and fixed in 70% ethanol for 2 h at 4°C and stained with RNase and PI reagent in darkness according to the Cell Cycle Analysis Kit (C1052, Beyotime) protocol. After incubation, the cells were analyzed by a flow cytometer.

### 4.13 MTT assay

The IMR‐90 cells were seeded 5000/well in 96‐well plates and treated with 1 μM doxorubicin for 24 h. 20 μl MTT (5 mg/ml) were added incubating at 37°C for 4 h, then dissolved in 150 μl DMSO and measured at 570 nm by microplate reader.

### 4.14 RNA sequencing

RNA was isolated from the WT, WT + SR9009, DOX (doxorubicin‐induced senescence), DOX + SR9009 cells using the Trizol (Invitrogen). Methods and Techniques for RNA sequencing were carried out by OE Biotech (Shanghai, China). Briefly, RNA integrity was evaluated by Agilent 2100 Bioanalyzer (Agilent Technologies, Santa Clara, CA, USA). TruSeq Stranded mRNA LTSample Prep Kit (Illumina, San Diego, CA, USA) was used to construct the libraries. Then, these libraries were sequenced on the Illumina sequencing platform (HiSeq X Ten), and 150 bp paired‐end reads were generated. Hisat2 (version 2.2.1.0) was used to calculate the RPKM (reads per kilobase per million) values through Cufflinks (version 2.2.1) and align the reads to the genome. GSEA was performed using GSEA 3.0 software from the Broad Institute (www.gseamsigdb.org). Gene ontology analysis was performed using DAVID functional annotation web resource (https://david‐d.ncifcrf.gov). Genes contributing to the top downregulated GO terms were combined with known SASP genes for heat map visualization.

### 4.15 Determination of Lifespan

Bristol N2 wild‐type were maintained and grown on Nematode Growth Medium (NGM) agar plates using E. coli OP50‐1 bacteria as a food source at 20°C. All NGM agar plates were prepared from the same batch and treatment plates were supplemented with the respective compounds DMSO, Rapamycin (100 μM), SR9009 (5 mM) or vehicle as a control. Fresh plates were prepared timely. FUDR was used to prevent reproduction. Worms were transferred to new plates and counted every other day. The lifespan was calculated with GraphPad Software.

### 4.16 Mice experiments

C57BL/6J (6–8 weeks) mice were housed in a specific‐pathogen‐free (SPF) facility. All related protocols were performed in compliance with the Guide for the Care and Use of Laboratory Animals and were approved by Institutional biomedical research ethics committee of Shanghai Institute of Nutrition and Health Sciences, Chinese Academy of Sciences. For ionizing radiation, mice were injected intraperitoneally SR9009 (100 mg/kg) every other day, then subjected to a sub‐lethal dose of 8 Gy irradiation, and organs were harvested 30 days after the procedure for immunoblots, immunohistochemistry, and SA‐β‐galactosidase staining. For oncogene‐induced senescence in liver, the transposon system encoding for oncogenic Nras^G12V^ and transposase‐encoding plasmid at 5:1 molar ratio (30 µg total DNA) was hydrodynamically injected via the tail vein. Livers were obtained on day 6 after injection for further analysis.

### 4.17 Statistical analysis

All experiments were performed using 3 independent repeated experiments for cells or 3–10 mice. The RNA‐seq data were obtained based on 2 independent biological replicates. All data are presented as means ± standard error of mean. Statistical analyses were calculated by the one‐way ANOVA and Student's *t*‐test to determine statistical significance. For all statistical tests, the 0.05 level of confidence was accepted for statistical significance.

## CONFLICT OF INTEREST

The authors have declared that no competing interest exists. Shanghai Institute of Nutrition and Health, Chinese Academy of Sciences has applied for a patent, China Patent Application No. 202011224266.6 by inventors Q.J., L.G., Z.L., Y.W. for the usage of SR9009 in anti‐senescence and the reduction of chronic inflammation caused by senescence described in this paper.

## AUTHOR CONTRIBUTIONS

Q.J. designed the experiments, supervised the project, and revised the manuscript. L.G., Z.L., Y.W., carried out experiments. Y.S., P.C., provided technical support. L.G. drafted the manuscript. All the authors approved the final manuscript.

## Supporting information

Supplementary Material

## Data Availability

The data that support the findings of this study are available in the supplementary file or from the corresponding author upon reasonable request.
