# Supplementary material for: Identification of a small molecule SR9009 that activates NRF2 to counteract cellular senescence
Source: Aging Cell. 2021 Sep 29;20(10):e13483. doi: 10.1111/acel.13483 (PMC8520720; doi:10.1111/acel.13483)
Supplement: Supplementary file 1 — Supplementary Material [file ACEL-20-e13483-s001.docx]

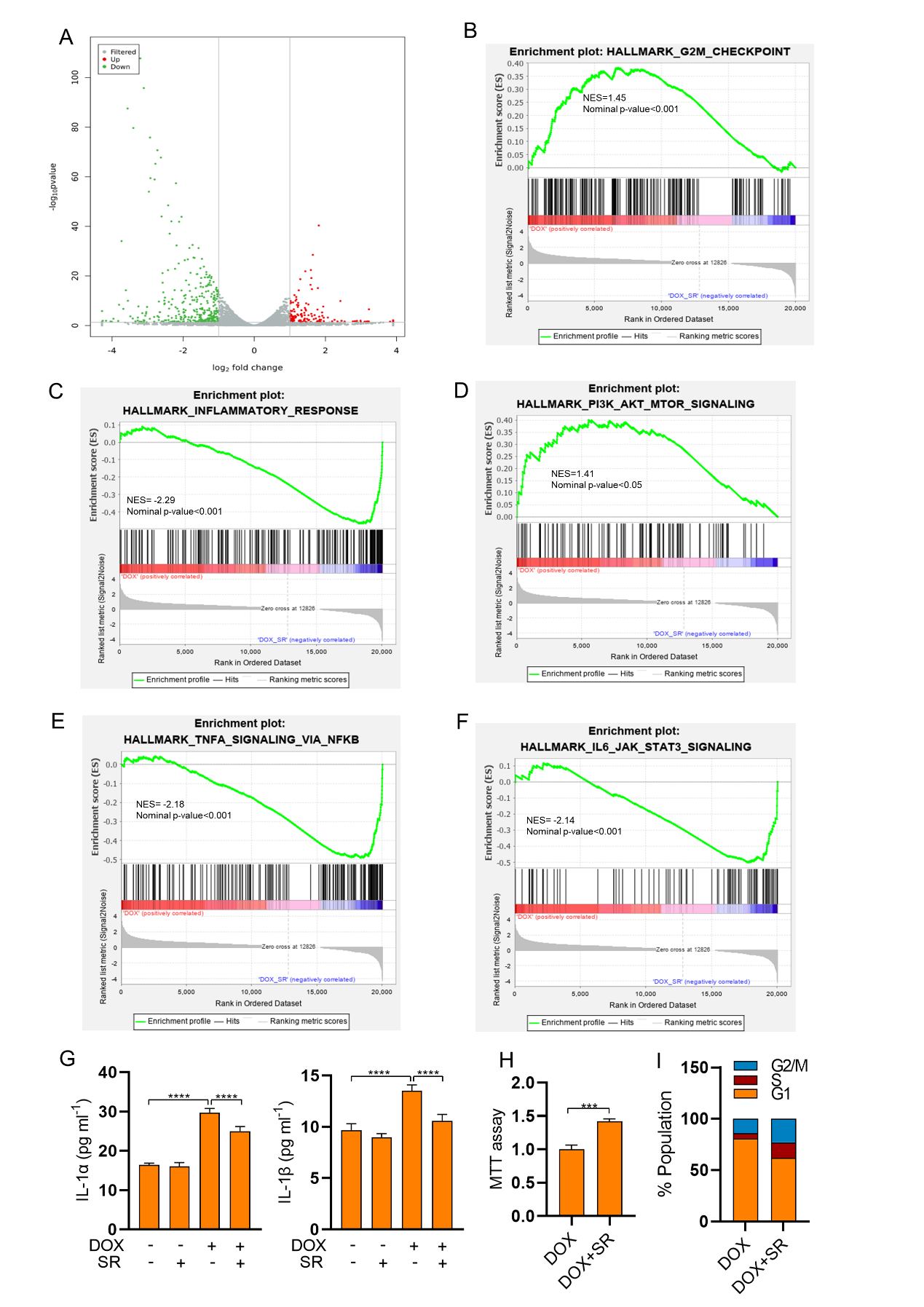


S**upplementary Figure 1. SR9009 suppresses inflammatory response and cell cycle arrest in HDFs undergoing doxorubicin-induced senescence.** (A) Volcano plot of differential expressed genes of DOX+SR9009 versus DOX HDFs. (B) GSEA enrichment plot of G2M CHECKPOINT in DOX+SR9009 HDFs. (C) GSEA enrichment plot of INFLAMMATORY RESPONSE in DOX+SR9009 HDFs. (D) GSEA enrichment plot of PI3K-AKT-MTOR SIGNALING in DOX+SR9009 HDFs. (E) GSEA enrichment plot of TNFA-SIGNALING-VIA-NF-ΚB in DOX+SR9009 HDFs. (F) GSEA enrichment plot of IL-6-JAK-STAT3 SIGNALING in DOX+SR9009 HDFs. (G) ELISA detection of IL-1α and IL-1β. (H) MTT assay of DOX, DOX+SR9009 HDFs. (I) Cell cycle analysis of DOX, DOX+SR9009 HDFs.


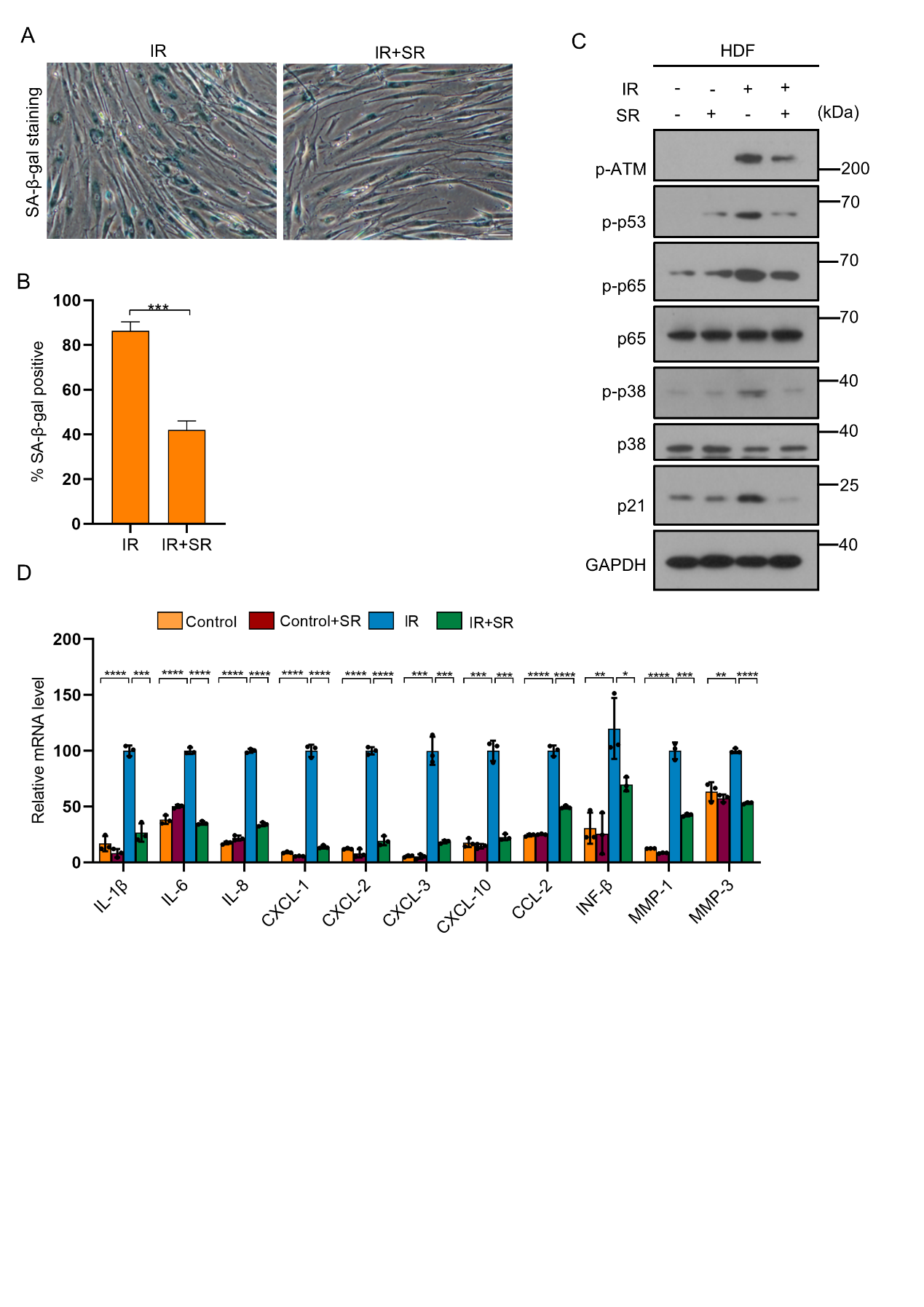


**Supplementary Figure 2. SR9009 attenuates the IR-induced senescence response.** (A) Senescence associated β-galactosidase (SA-β-Gal) of Control, Control+SR9009, IR (irradiation), IR+SR9009 HDFs. Scale bar, 50 μm. (B) Quantitative analysis of SA-β-Gal positive cells of HDFs in different groups. (C) Western blot analysis of DNA damage factor p-ATM, p-p53, cell arrest factor p21, transcription factor p-p38, p-p65 regulating the expression of SASP in different groups. GAPDH was used as loading control. The representative data from three independent experiments are shown. (D) RT-qPCR analysis of SASP factor gene expression, RPL13A was used as loading control. For all graphs, error bars indicate mean ± SEM of triplicate measurements. *P < 0.05, **P < 0.01, ***P < 0.001; Student’s t-test (B) and one-way ANOVA for all others.


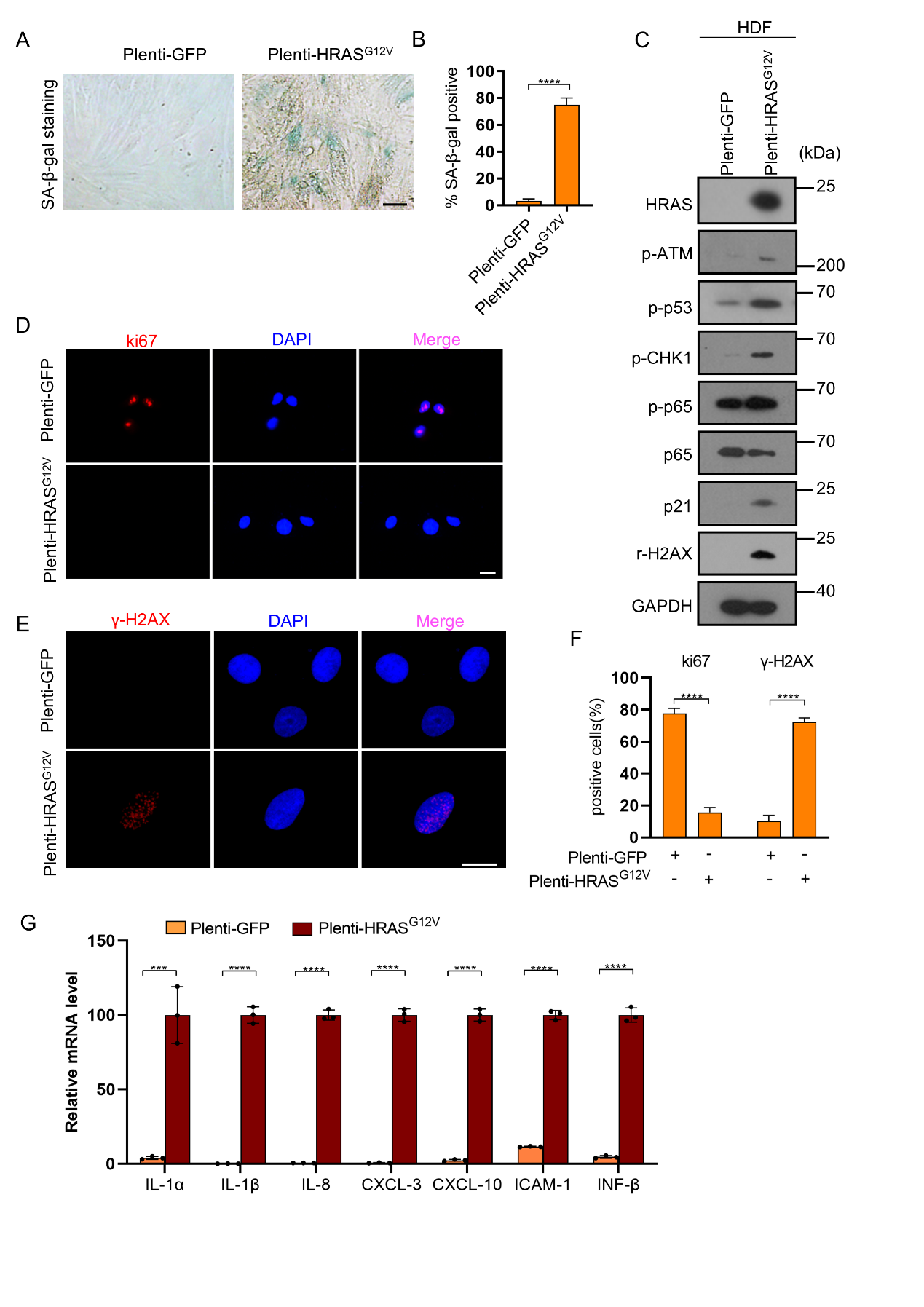


**Supplementary Figure 3. Overexpression of HRAS^G12V^ induces the senescence of HDFs.** (A) SA-β-Gal staining the HDFs undergoing oncogene-induced senescence. Scale bar, 50 μm. (B) Quantitative analysis of SA-β-Gal positive cells. (C) Western blot detection of the expression change of DNA damage markers and the key pathway regulating the SASP in HDFs undergoing oncogene-induced senescence. (D) Immunofluorescence staining for heterochromatin marker (H3KeMe3[red], and 40,6-diamidino-2-phenylindole [blue]). Scale bar, 20 μm. (E) immunofluorescence staining for DNA damage foci (γ-H2AX [red], and 40,6-diamidino-2-phenylindole [blue]). Scale bar, 20 μm. (F) Quantitative analysis of Immunofluorescence staining results. (G) RT-qPCR analysis of SASP factor gene expression, RPL13A was used as loading control. The representative data from three independent experiments are shown. For all graphs, error bars indicate mean ± SEM of triplicate measurements. *P < 0.05, **P < 0.01, ***P < 0.001, ****P < 0.0001; Student’s t-test (B) and one-way ANOVA for all others.


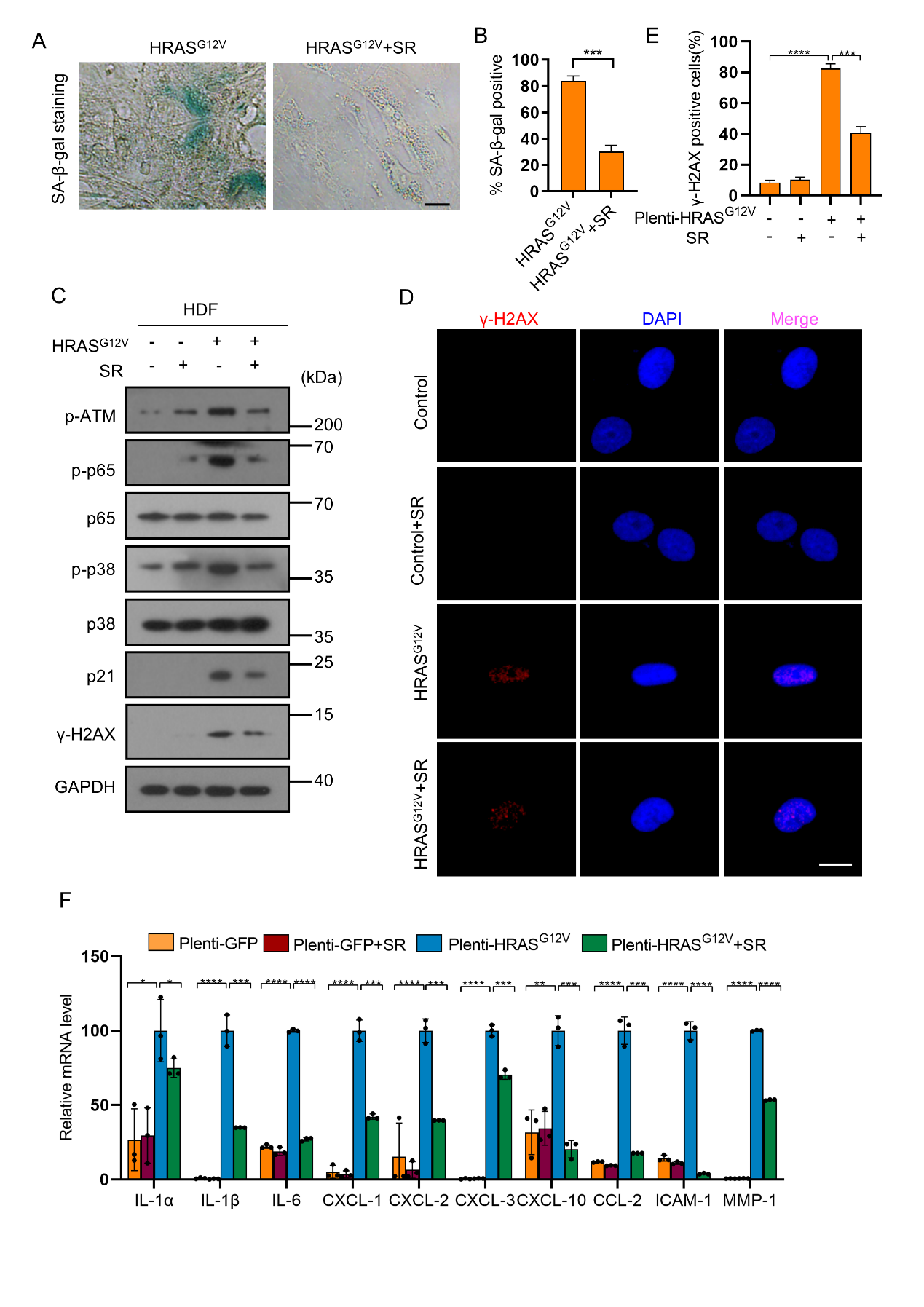


**Supplementary Figure 4. SR9009 suppresses the senescence response of HDFs undergoing oncogene-induced senescence.** (A) Senescence associated β-galactosidase (SA-β-Gal) of HDFs undergoing oncogene-induced senescence in the absence or presence of SR9009. Scale bar, 50 μm. (B) Quantitative analysis of SA-β-Gal positive cells of HDFs. (C) Western blot analysis of DNA damage factor p-ATM, cell arrest factor p21, transcription factor p-p38, p-p65 regulating the expression of SASP. GAPDH was used as loading control. (D) Immunofluorescence staining for marker of DNA damage (γ-H2AX [red], and 40,6-diamidino-2-phenylindole [blue]) after SR9009 treatment. Scale bar, 20 μm. (E) Quantitative analysis of Immunofluorescence staining results. (F) RT-qPCR analysis of SASP factor gene expression, RPL13A was used as loading control. The representative data from three independent experiments are shown. For all graphs, error bars indicate mean ± SEM of triplicate measurements. *P < 0.05, **P < 0.01, ***P < 0.001, ****P < 0.0001; Student’s t-test (B) and one-way ANOVA for all others.


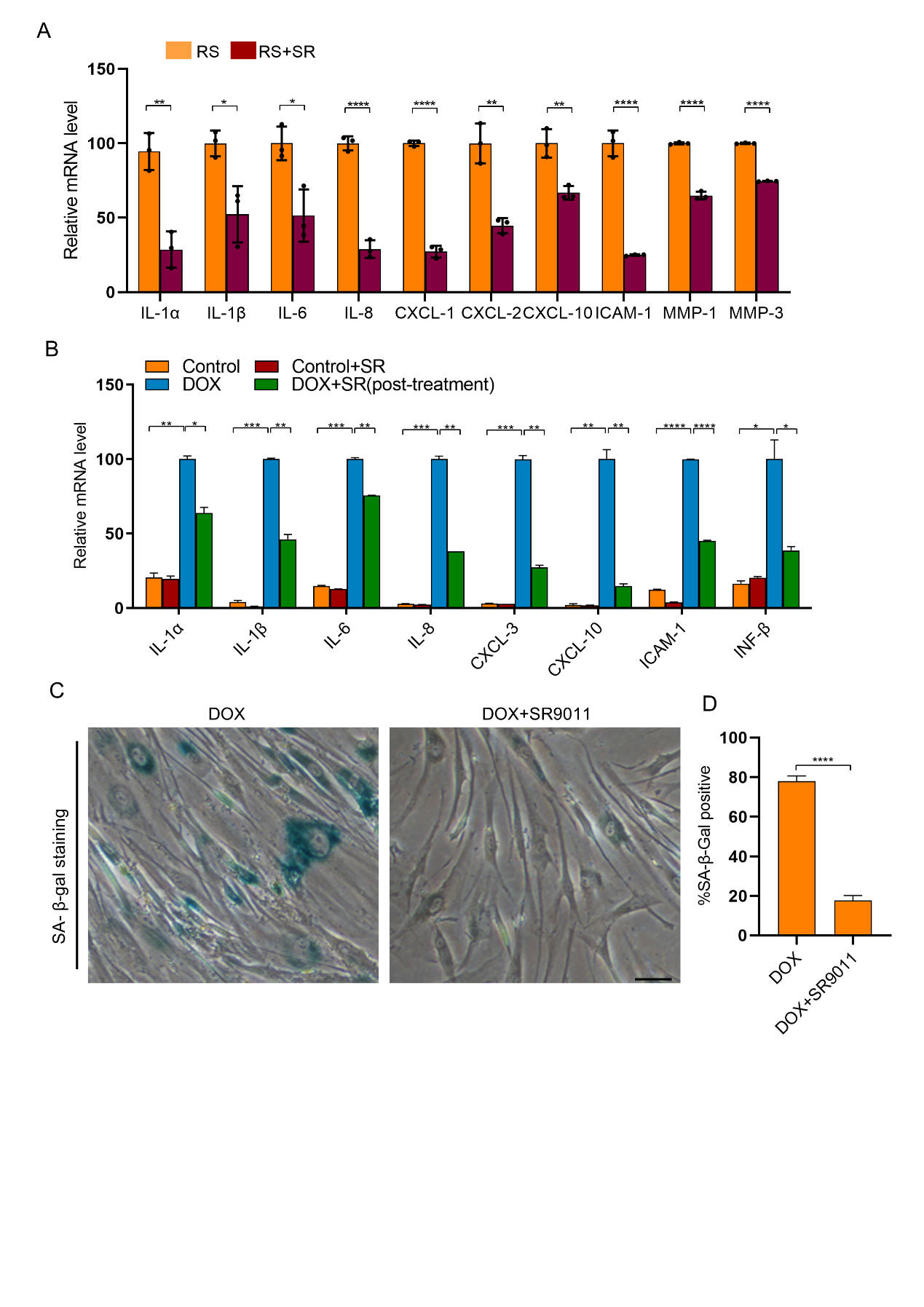


**Supplementary Figure 5. SR9009 and SR9011 suppresses the senescence phenotype of HDFs.** (A) Replicative senescence HDFs were added SR9009 and detected the expression of SASP factors by RT-qPCR. (B) HDFs were induced senescence by doxorubicin, then were added SR9009 and detected the expression of SASP factors by RT-qPCR. RPL13A was used as loading control. The representative data from three independent experiments are shown. (C) Senescence associated β-galactosidase (SA-β-Gal) of HDFs undergoing chemotherapy-induced senescence in the absence or presence of SR9011. Scale bar, 50 μm. For all graphs, error bars indicate mean ± SEM of triplicate measurements. *P < 0.05, **P < 0.01, ***P < 0.001, ****P < 0.0001; Student’s t-test (A, C) and one-way ANOVA for all others.


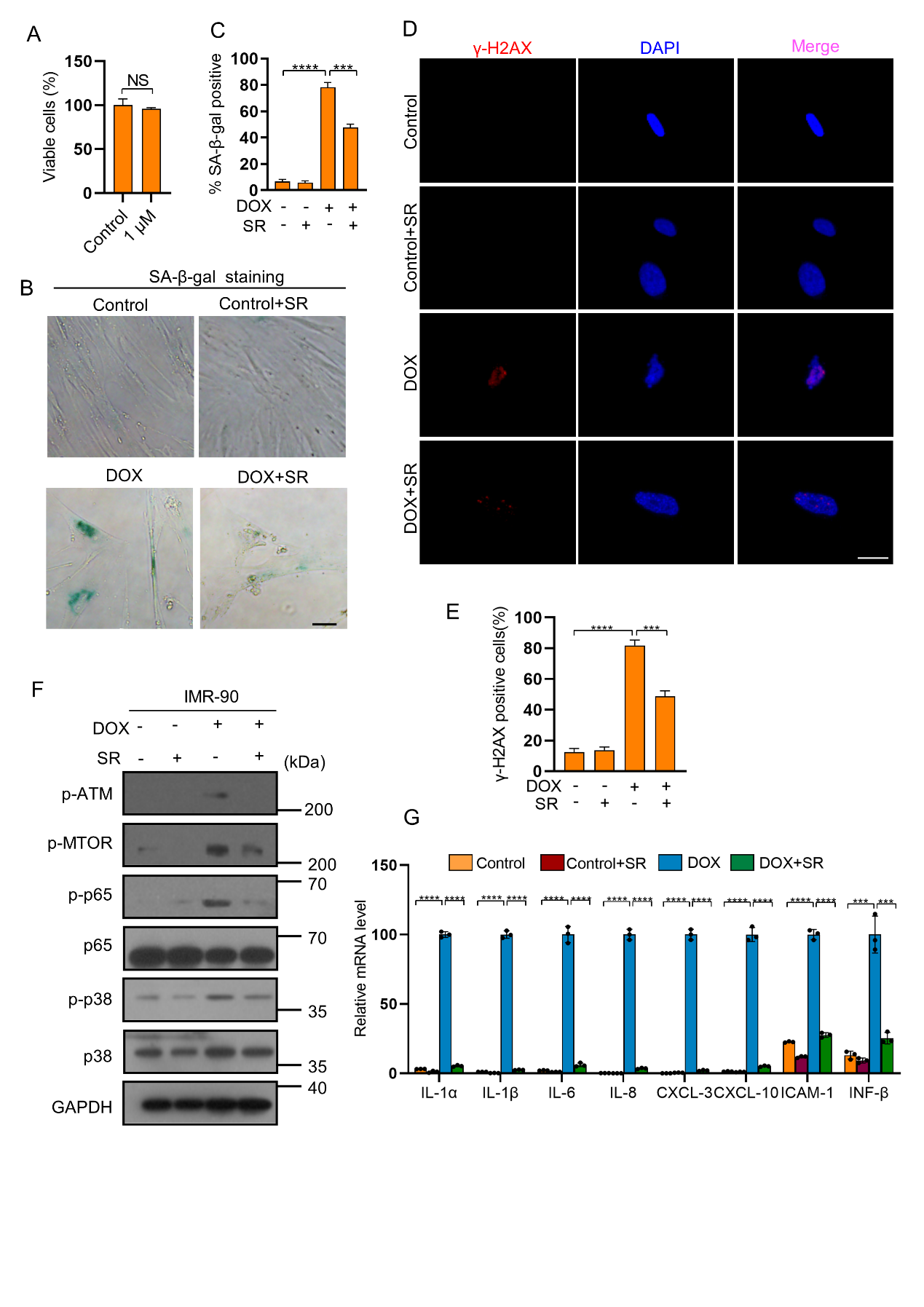


**Supplementary Figure 6. SR9009 suppresses the senescence response of IMR90s undergoing chemotherapy-induced senescence.** (A) HDFs were treated with 1 uM doxorubicin 24h and cell viability were measured by MTT. (B) Senescence associated β-galactosidase (SA-β-Gal) of Control, Control+SR9009, DOX (doxorubicin), DOX+SR9009 IMR90s. Scale bar, 50 μm. (C) Quantitative analysis of SA-β-Gal positive cells of IMR90s in different groups. (D) Immunofluorescence staining for marker of DNA damage (γ-H2AX [red], and 40,6-diamidino-2-phenylindole [blue]) upon SR9009 treatment or not. Scale bar, 20 μm. (E) Quantitative analysis of Immunofluorescence staining results. (F) Western blot analysis of DNA damage factor p-ATM, p-MTOR, transcription factor p-p38, p-p65 regulating the expression of SASP. GAPDH was used as loading control. (G) RT-qPCR analysis of SASP factor gene expression in different groups, RPL13A was used as loading control. The representative data from three independent experiments are shown. For all graphs, error bars indicate mean ± SEM of triplicate measurements. *P < 0.05, **P < 0.01, ***P < 0.001, ****P < 0.0001; one-way ANOVA.


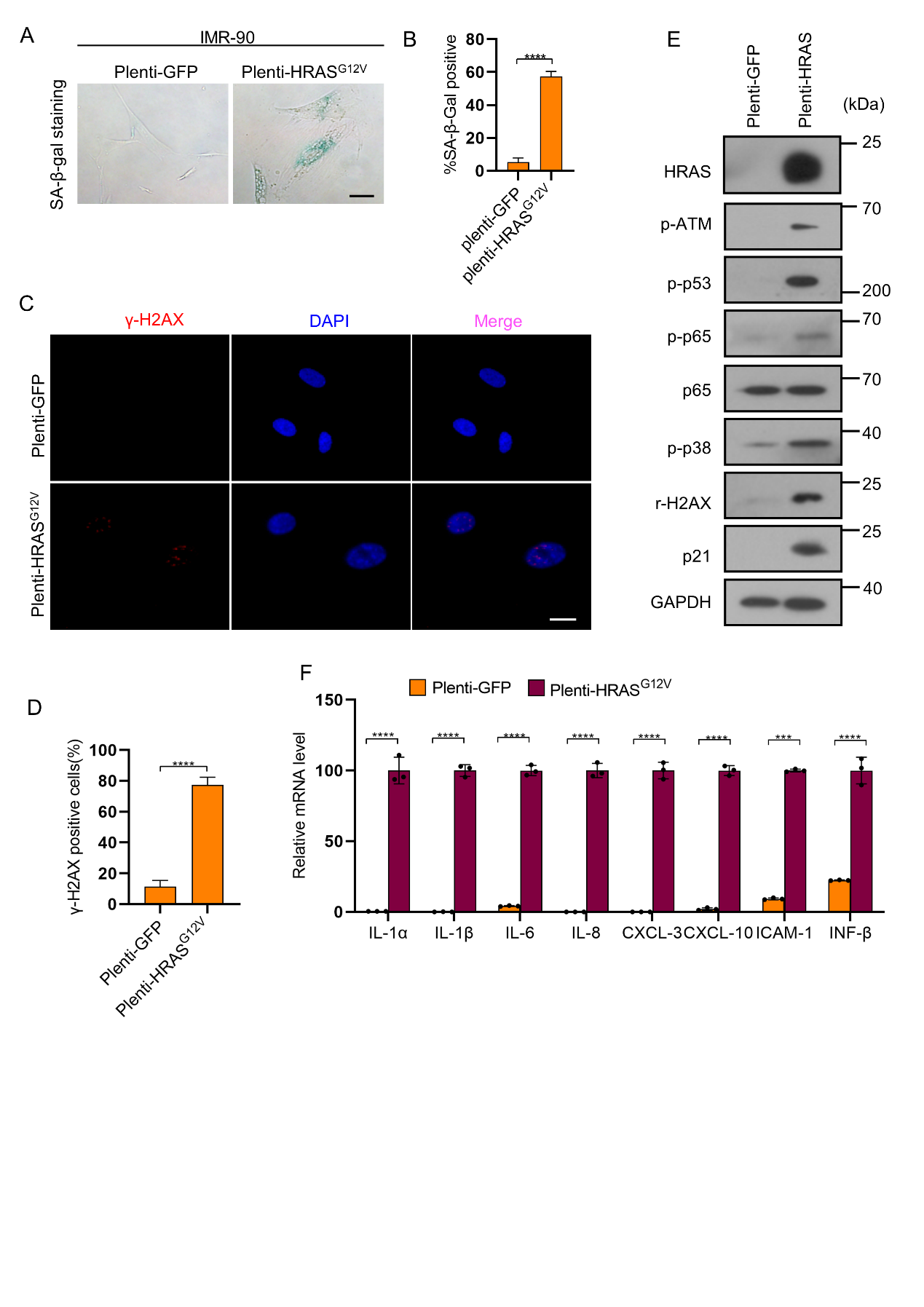
**Supplementary Figure 7. Overexpression of HRAS^G12V^ causes the senescence response of IMR90s.** (A) SA-β-Gal staining of Control, HRAS^G12V^ IMR90s. Scale bar, 50 μm. (B) Quantitative analysis of SA-β-Gal positive cells of IMR90s in different groups. (C) Overexpression of HRAS^G12V^ accumulated the DDR foci of IMR90s determined by immunofluorescence staining of γ-H2AX. Scale bar, 20 μm. (D) Quantitative analysis of Immunofluorescence staining results. (E) Western blot analysis of the expression of DNA damage factor p-ATM, γ-H2AX, p-p53, cell arrest factor p21, and transcription factor p-p38, p-p65 regulating the expression of SASP induced by HRASG12V. GAPDH was used as loading control. (F) RT-qPCR analysis of SASP factor gene expression in different groups, RPL13A was used as loading control. Graphs show mean values with SEM; *P < 0.05, **P < 0.01, ***P < 0.001, ****P < 0.0001; Student’s t-test (B) and one-way ANOVA for all others.


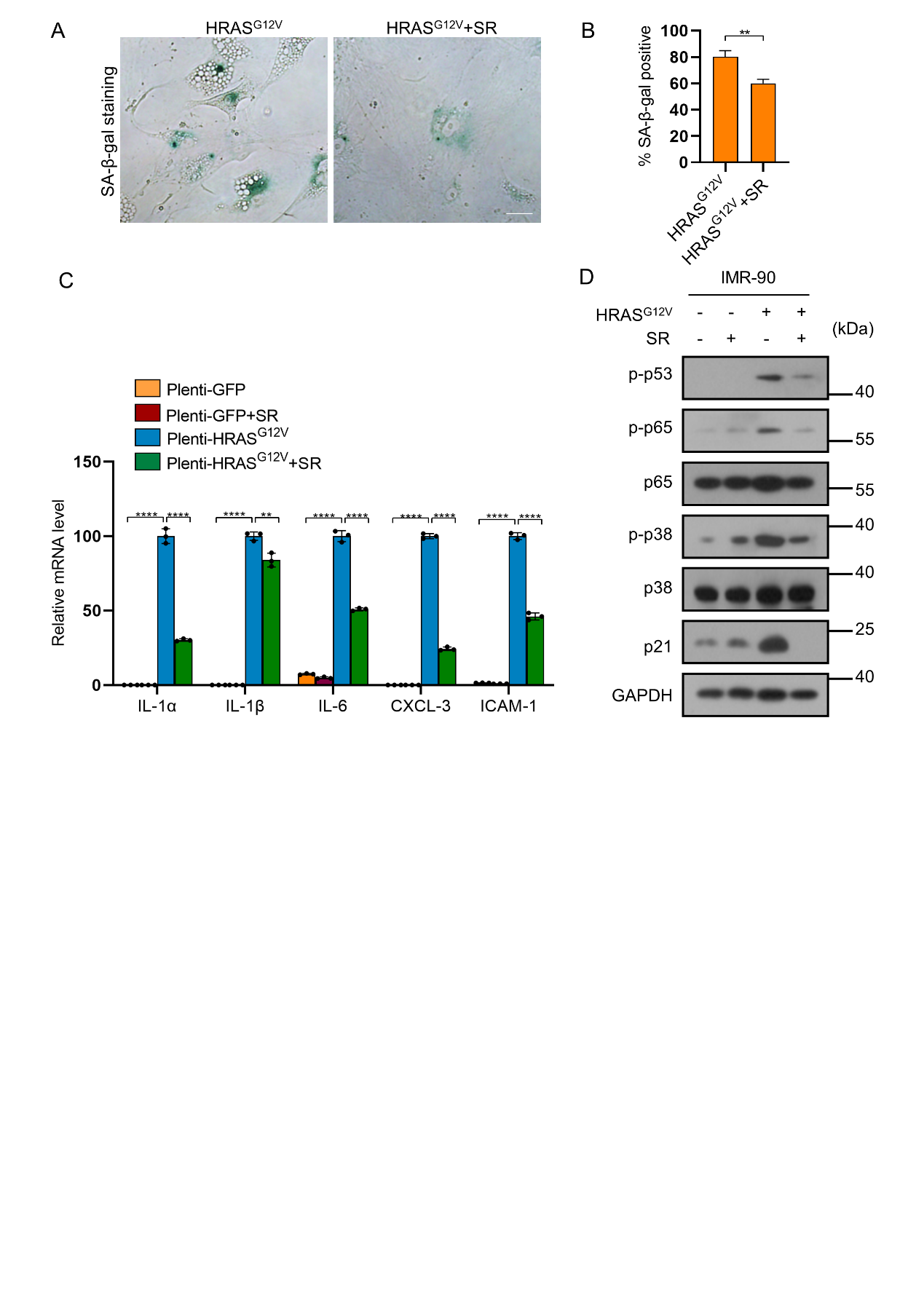


**Supplementary Figure 8. SR9009 alleviates the senescence response of IMR90s undergoing oncogene-induced senescence.** (A) SA-β-Gal staining of HRAS^G12V^, HRAS^G12V^+SR9009 IMR90s. Scale bar, 50 μm. (B) Quantitative analysis of SA-β-Gal positive cells of IMR90s in different groups. (C) RT-qPCR analysis of SASP factor gene expression, RPL13A was used as loading control. (D) Western blot analysis of DNA damage factor p-p53, cell arrest factor p21, transcription factor p-p38, p-p65 regulating the expression of SASP. GAPDH was used as loading control. The representative data from three independent experiments are shown. For all graphs, error bars indicate mean ± SEM of triplicate measurements. *P < 0.05, **P < 0.01, ***P < 0.001, ****P < 0.0001; Student’s t-test (B) and one-way ANOVA for all others.


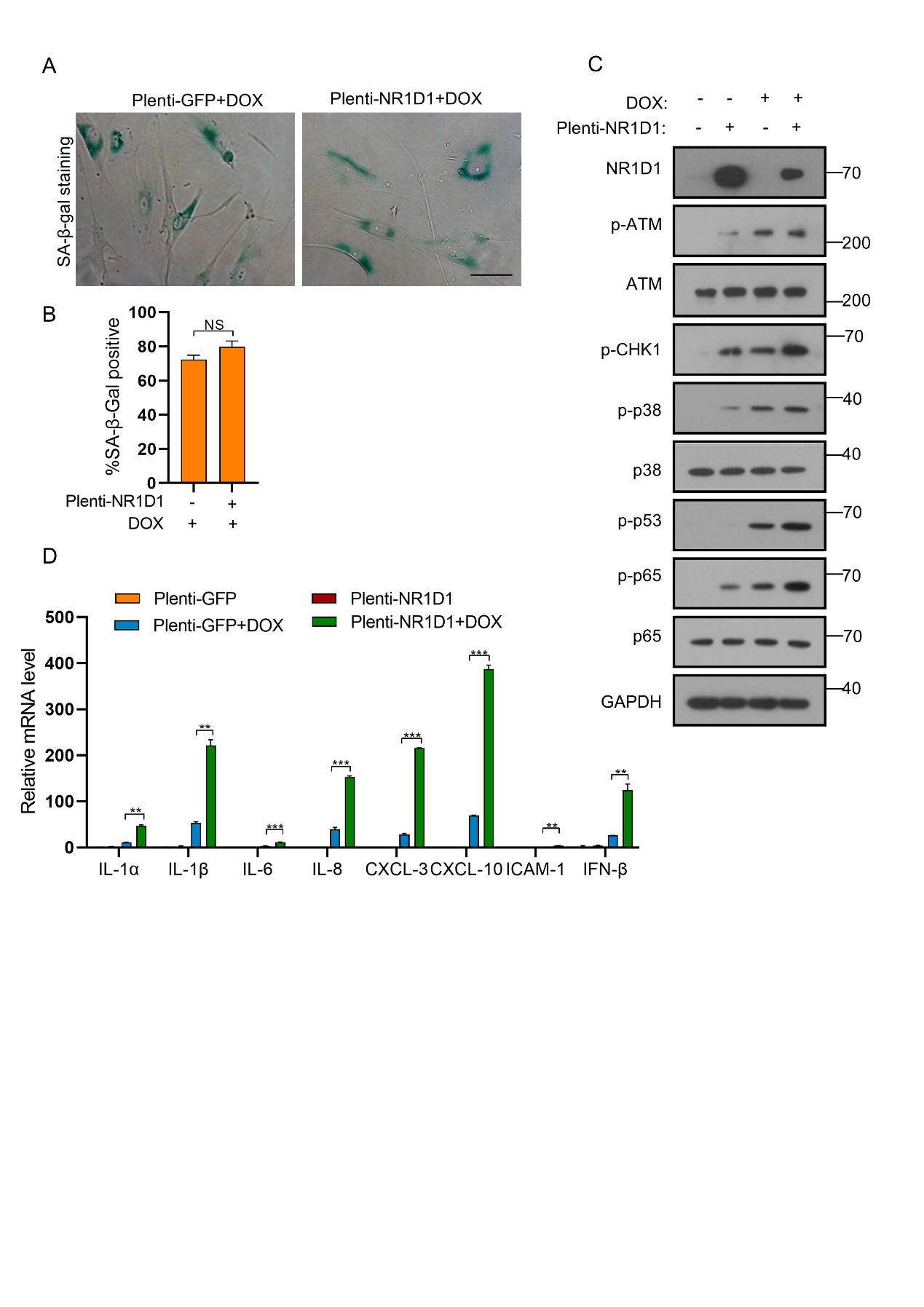


**Supplementary Figure 9. Overexpression of NR1D1 aggravates the senescence phenotypes of HDFs undergoing chemotherapy-induced senescence response.** (A) SA-β-Gal staining of Plenti-GFP, Plenti-NR1D1 group after chemotherapy-induced senescence. Scale bar, 50 μm. (B) Quantitative analysis of SA-β-Gal positive cells described in A. (C) Western blot analysis of DNA damage factor p-p53, cell arrest factor p21, transcription factor p-p38, p-p65 regulating the expression of SASP. GAPDH was used as loading control. (D) RT-qPCR analysis of SASP factor gene expression, RPL13A was used as loading control. For all graphs, error bars indicate mean ± SEM of triplicate measurements. *P < 0.05, **P < 0.01, ***P < 0.001, ****P < 0.0001; Student’s t-test (B) and one-way ANOVA for all others


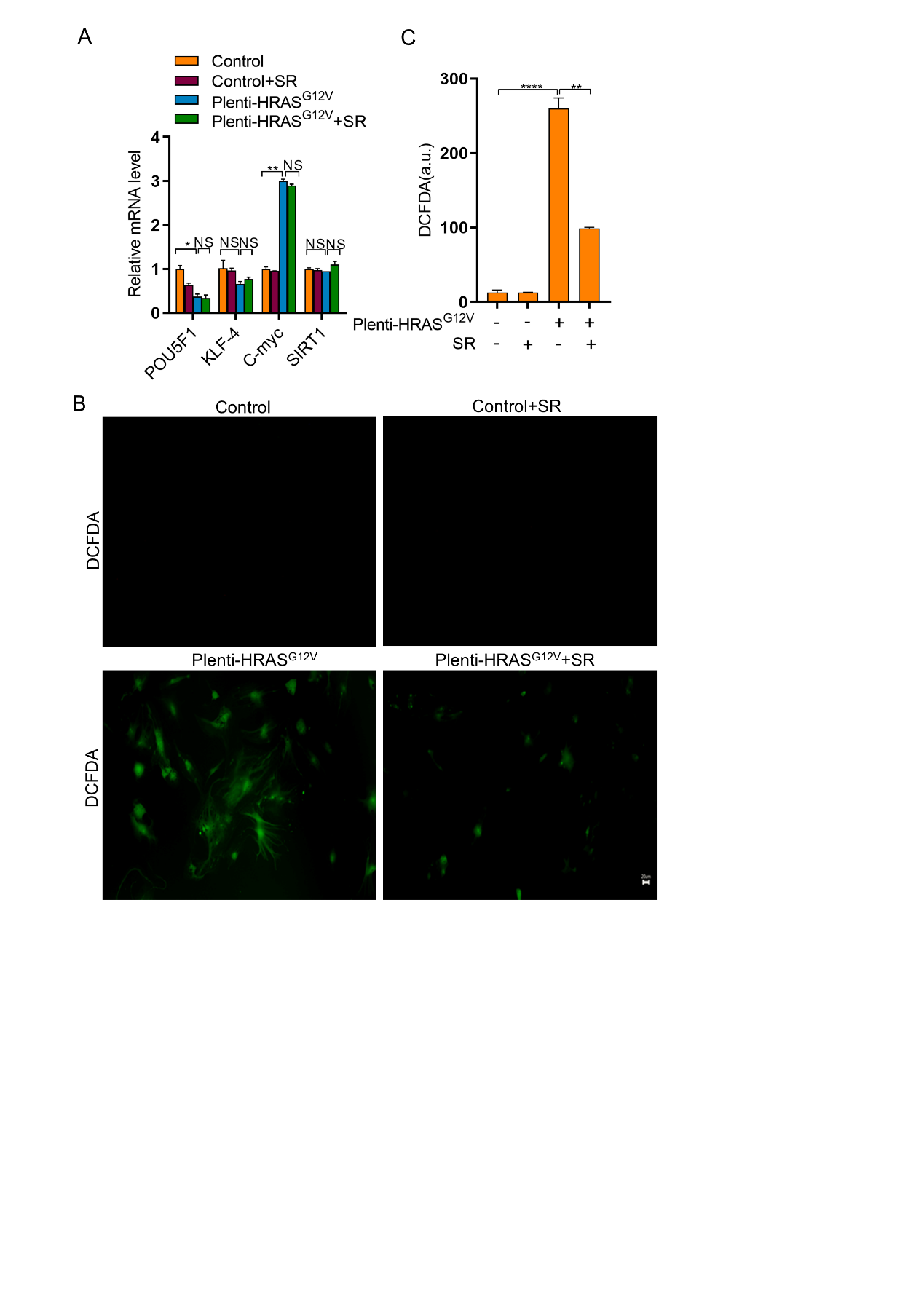


**Supplementary Figure 10. SR9009 decreases the ROS level of HDF cells undergoing oncogene-induced senescence response.** (A) RT-qPCR detection of the expression of pluripotency related genes and SIRT1 in HDFs undergoing oncogene-induced senescence after SR9009 treatment. RPL13A was used as loading control. (B) Analysis of reactive oxygen species (ROS) level by H2DCF-DA staining in HDFs pretreated or not (control) with SR9009. Scale bar, 20 μm. (C) Quantitative analysis of the fluorescence intensity stained by H2DCF-DA. *P < 0.05, **P < 0.01, ***P < 0.001, ****P < 0.0001; Student’s t-test (B) and one-way ANOVA for all others.


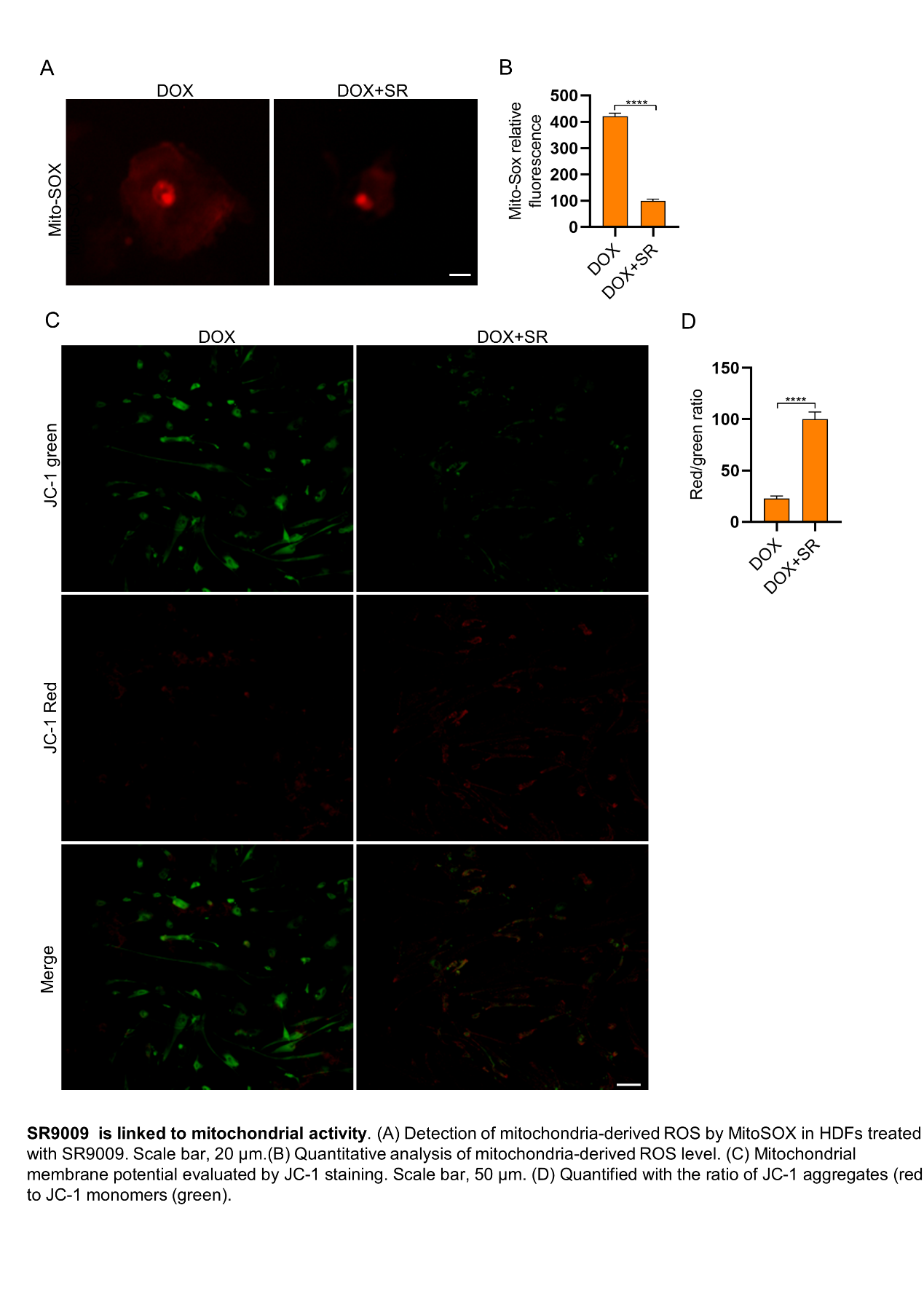


**Supplementary Figure 11. SR9009 is linked to** **mitochondrial activity.** (A) Detection of mitochondria-derived ROS by MitoSOX in HDFs treated with SR9009. Scale bar, 20 μm. (B) Quantitative analysis of mitochondria-derived ROS level. (C) Mitochondrial membrane potential is evaluated by JC-1 staining. Scale bar, 50 μm. (D) Quantified with the ratio of JC-1 aggregates (red) to JC-1 monomers (green).


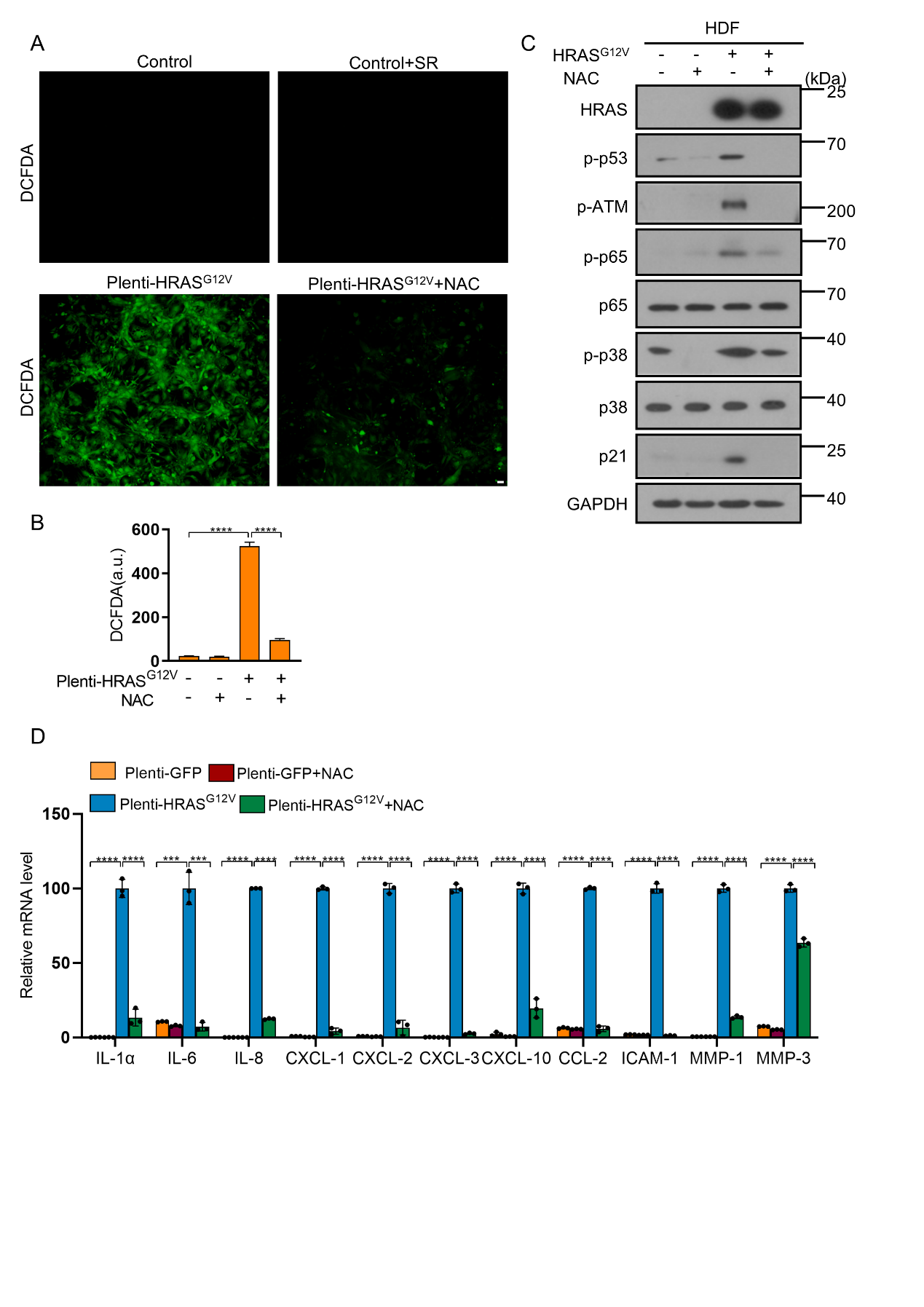


**Supplementary Figure 12. NAC reverses the senescence response of HDFs undergoing oncogene-induced senescence response.** (A) Analysis of reactive oxygen species (ROS) level by H2DCF-DA staining in HDFs induced by HRAS^G12V^ in the presence or absent NAC. Scale bar, 20 μm. (B) Quantitative analysis of the fluorescence intensity stained by H2DCF-DA. (C) Western blot analysis of DNA damage factor p-ATM, p-p53, cell arrest factor p21, transcription factor p-p38, p-p65 regulating the expression of SASP. GAPDH was used as loading control. (D) RT-qPCR analysis of SASP factor gene expression of HDFs after NAC treatment. RPL13A was used as loading control. The representative data from three independent experiments are shown. For all graphs, error bars indicate mean ± SEM of triplicate measurements. *P < 0.05, **P < 0.01, ***P < 0.001, ****P < 0.0001; Student’s t-test (B) and one-way ANOVA for all others.


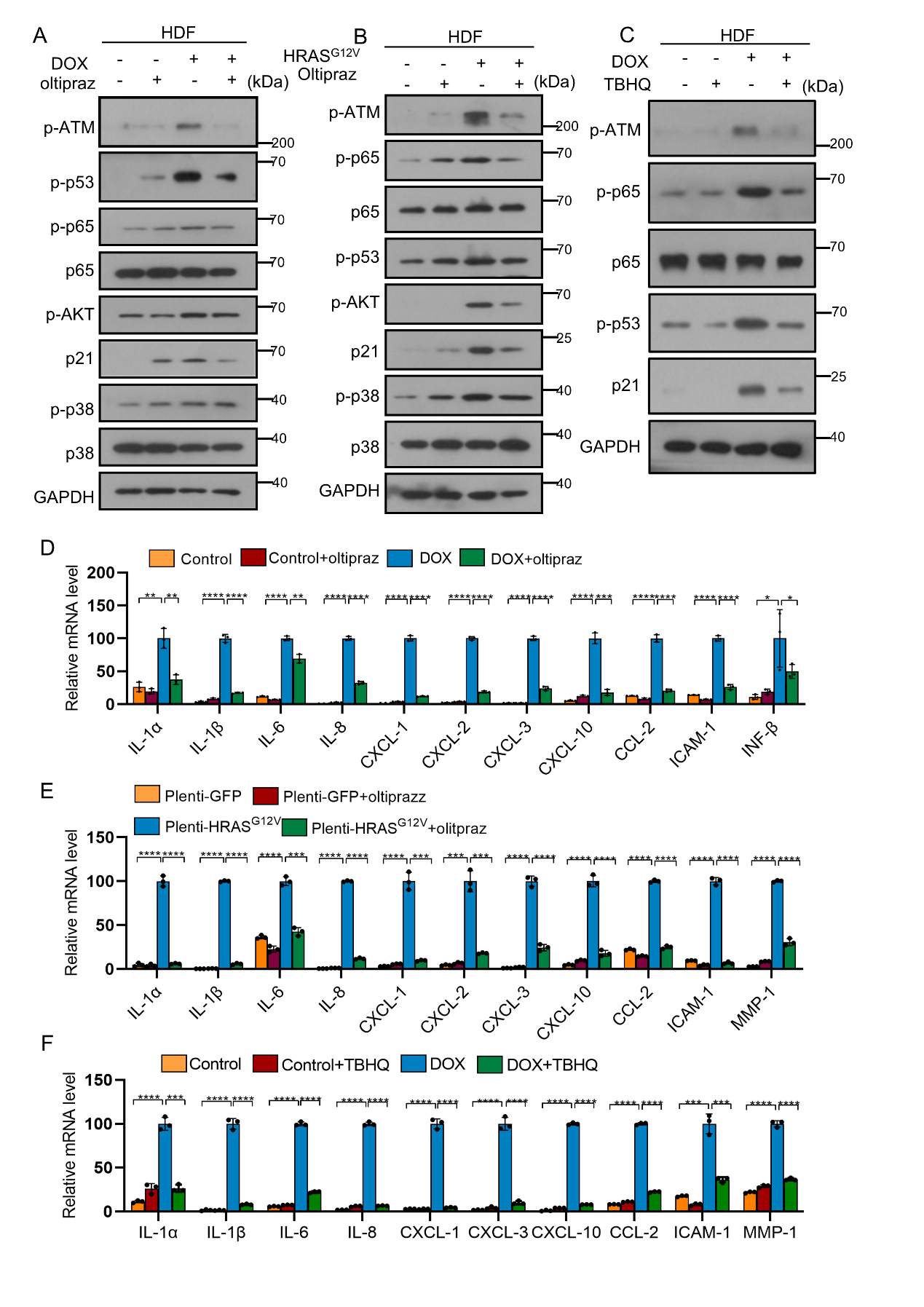


**Supplementary Figure 13. The nuclear accumulation of NRF2 suppresses the** **senescence phenotype of HDFs.** (A) HDFs were pretreated by oltipraz, then undergone senescence induced by doxorubicin. These cells were subjected to western blotting analysis using indicated antibodies shown left. GAPDH was used as loading control. (B) HDFs were pretreated by oltipraz, then undergone senescence induced by HRAS^G12V^. These cells were subjected to western blotting analysis using indicated antibodies shown left. GAPDH was used as loading control. (C) HDFs were pretreated by TBHQ, then undergone senescence induced by doxorubicin. These cells were subjected to western blotting analysis using indicated antibodies shown left. GAPDH was used as loading control. (D) RT-qPCR analysis of SASP factor gene expression described in (A). RPL13A was used as loading control. (E) RT-qPCR analysis of SASP factor gene expression described in (B). RPL13A was used as loading control. (F) RT-qPCR analysis of SASP factor gene expression described in (C). RPL13A was used as loading control. For all graphs, error bars indicate mean ± SEM of triplicate measurements. *P < 0.05, **P < 0.01, ***P < 0.001, ****P < 0.0001; one-way ANOVA.


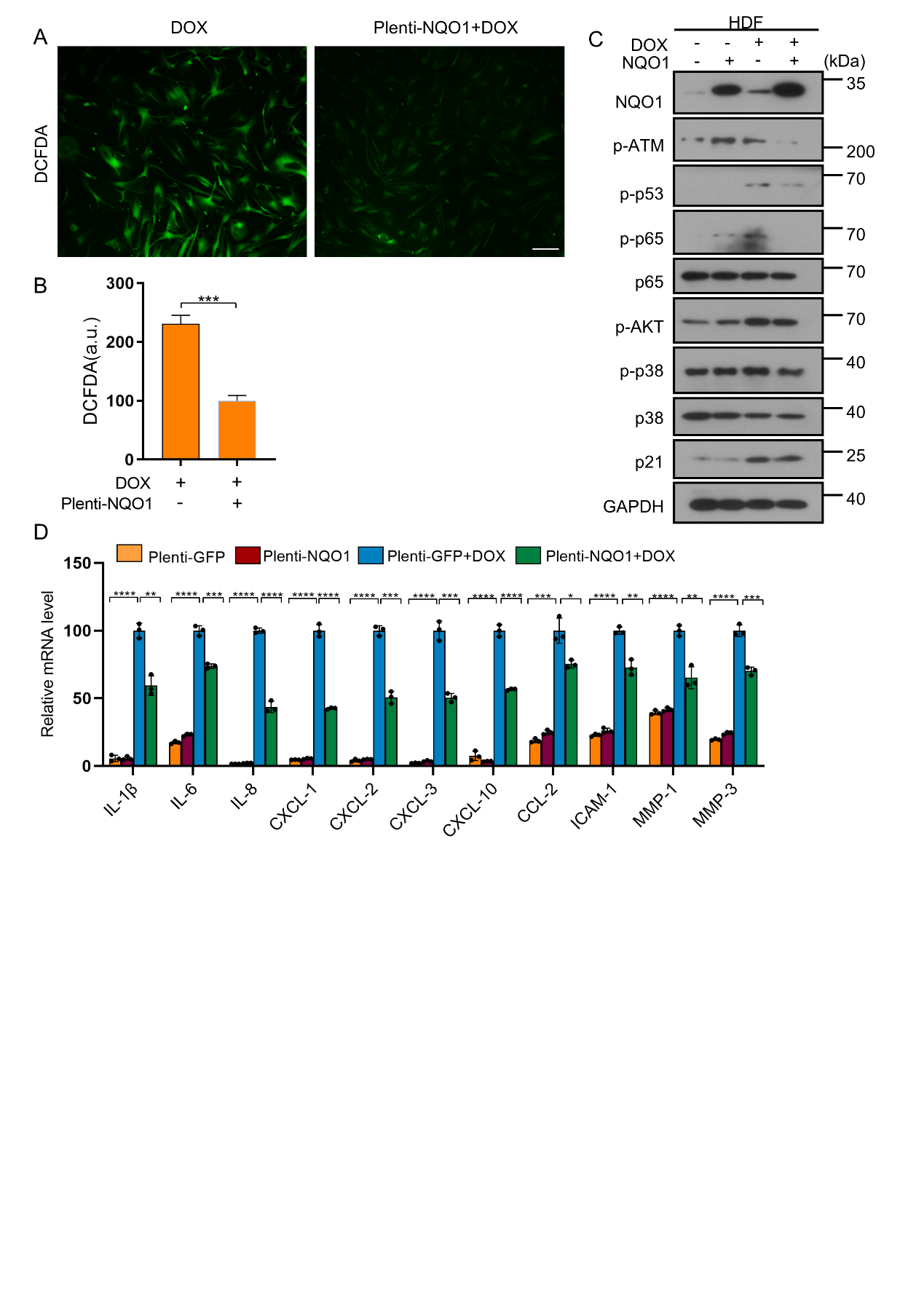


**Supplementary Figure 14. Overexpression of NQO1 alleviates the senescence response of HDFs undergoing chemotherapy-induced senescence response.** (A) DCFDA-based ROS quantification in HDF cells overexpressing HO-1 or control cells undergoing doxorubicin-induced senescence. (B) Quantitative analysis of reactive oxygen species (ROS) level. (C) Western blot analysis of DNA damage factor p-ATM, p-p53, cell arrest factor p21, transcription factor p-p38, p-p65 regulating the expression of SASP. GAPDH was used as loading control. (D) RT-qPCR analysis of SASP factor gene expression of HDFs after overexpression of NQO1. The representative data from three independent experiments are shown. For all graphs, error bars indicate mean ± SEM of triplicate measurements. *P < 0.05, **P < 0.01, ***P < 0.001, ****P < 0.0001; Student’s t-test (B) and one-way ANOVA for all others.


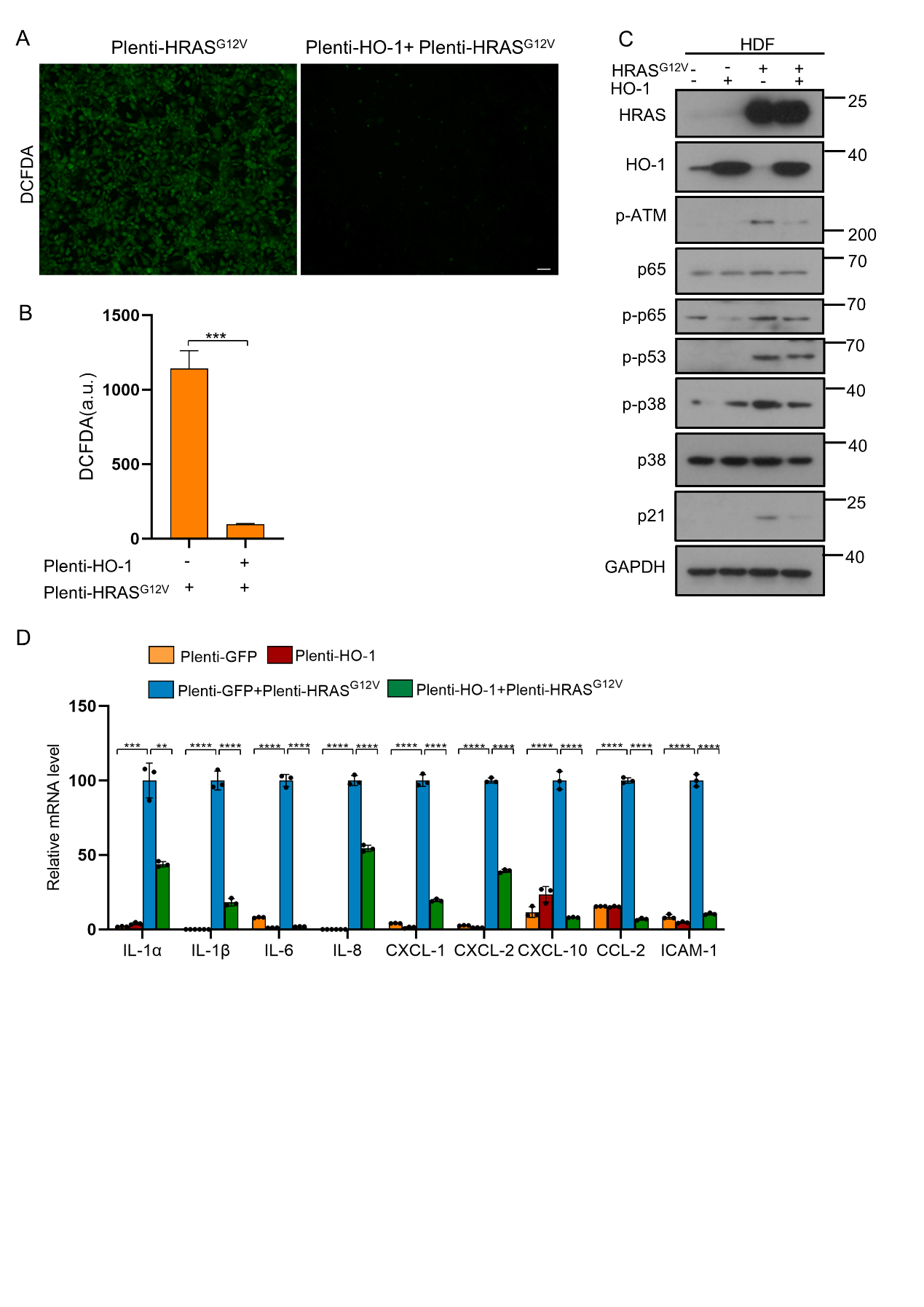


**Supplementary Figure 15. Overexpression of HO-1 prevents the senescence response of HDFs undergoing oncogene-induced senescence response.** (A) DCFDA-based ROS quantification in HDF cells overexpressing HO-1 or control cells undergoing oncogene-induced senescence. Scale bar, 50 μm. (B) Quantitative analysis of reactive oxygen species (ROS) level. (C) Western blot analysis of DNA damage factor p-ATM, p-p53, cell arrest factor p21, transcription factor p-p38, p-p65 regulating the expression of SASP. GAPDH was used as loading control. (D) RT-qPCR analysis of SASP factor gene expression of HDFs after overexpression of HO-1. The representative data from three independent experiments are shown. For all graphs, error bars indicate mean ± SEM of triplicate measurements. *P < 0.05, **P < 0.01, ***P < 0.001, ****P < 0.0001; Student’s t-test (B) and one-way ANOVA for all others.


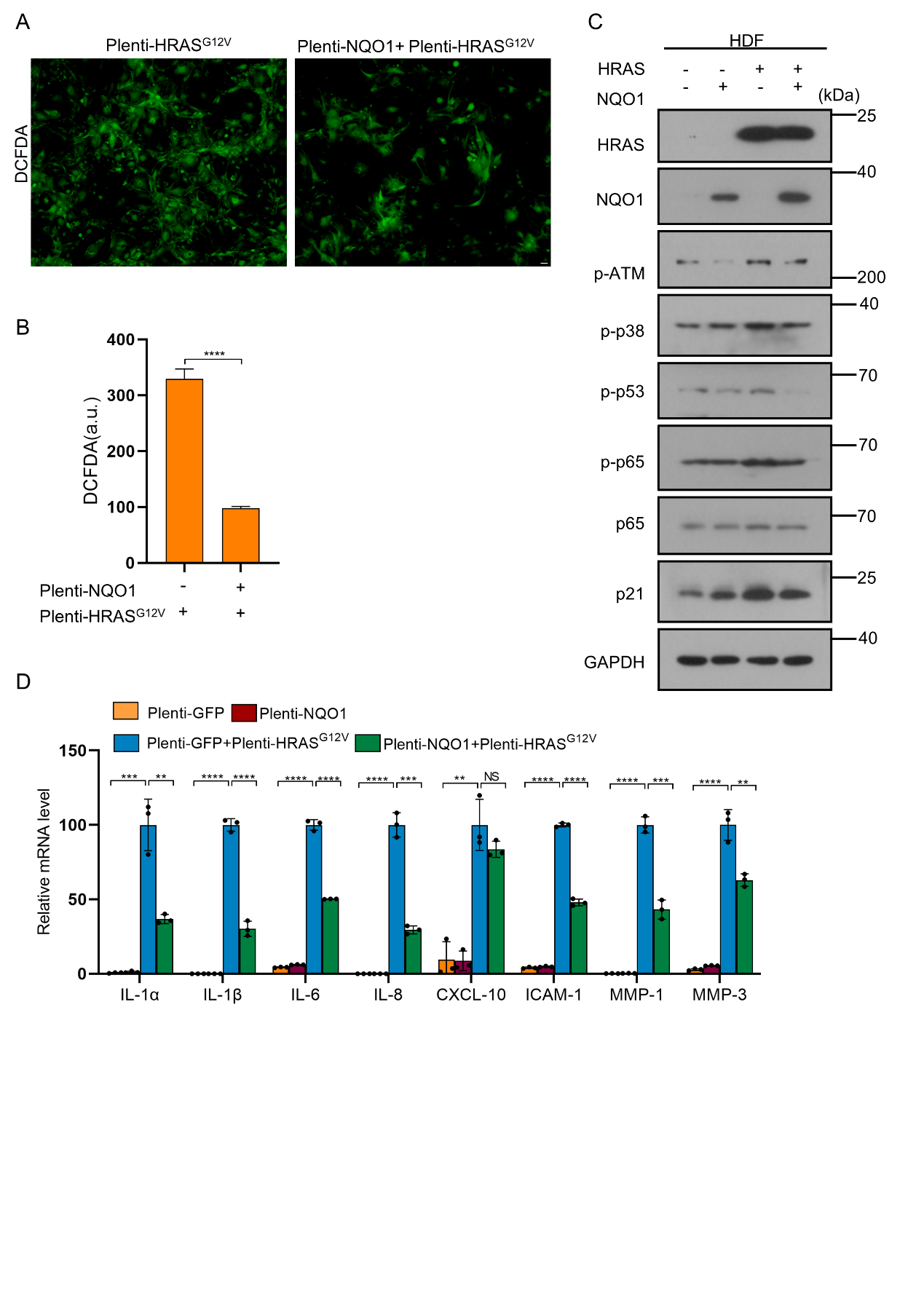


**Supplementary Figure 16. Overexpression of NQO1 alleviates the senescence response of HDFs undergoing oncogene-induced senescence response.** (A) DCFDA-based ROS quantification in HDF cells overexpressing NQO1 or control cells undergoing oncogene-induced senescence. Scale bar, 20 μm. (B) Quantitative analysis of reactive oxygen species (ROS) level. (C) Western blot analysis of DNA damage factor p-ATM, p-p53, cell arrest factor p21, transcription factor p-p38, p-p65 regulating the expression of SASP. GAPDH was used as loading control. (D) RT-qPCR analysis of SASP factor gene expression of HDFs after overexpression of NQO1. The representative data from three independent experiments are shown. For all graphs, error bars indicate mean ± SEM of triplicate measurements. *P < 0.05, **P < 0.01, ***P < 0.001, ****P < 0.0001; Student’s t-test (B) and one-way ANOVA for all others.


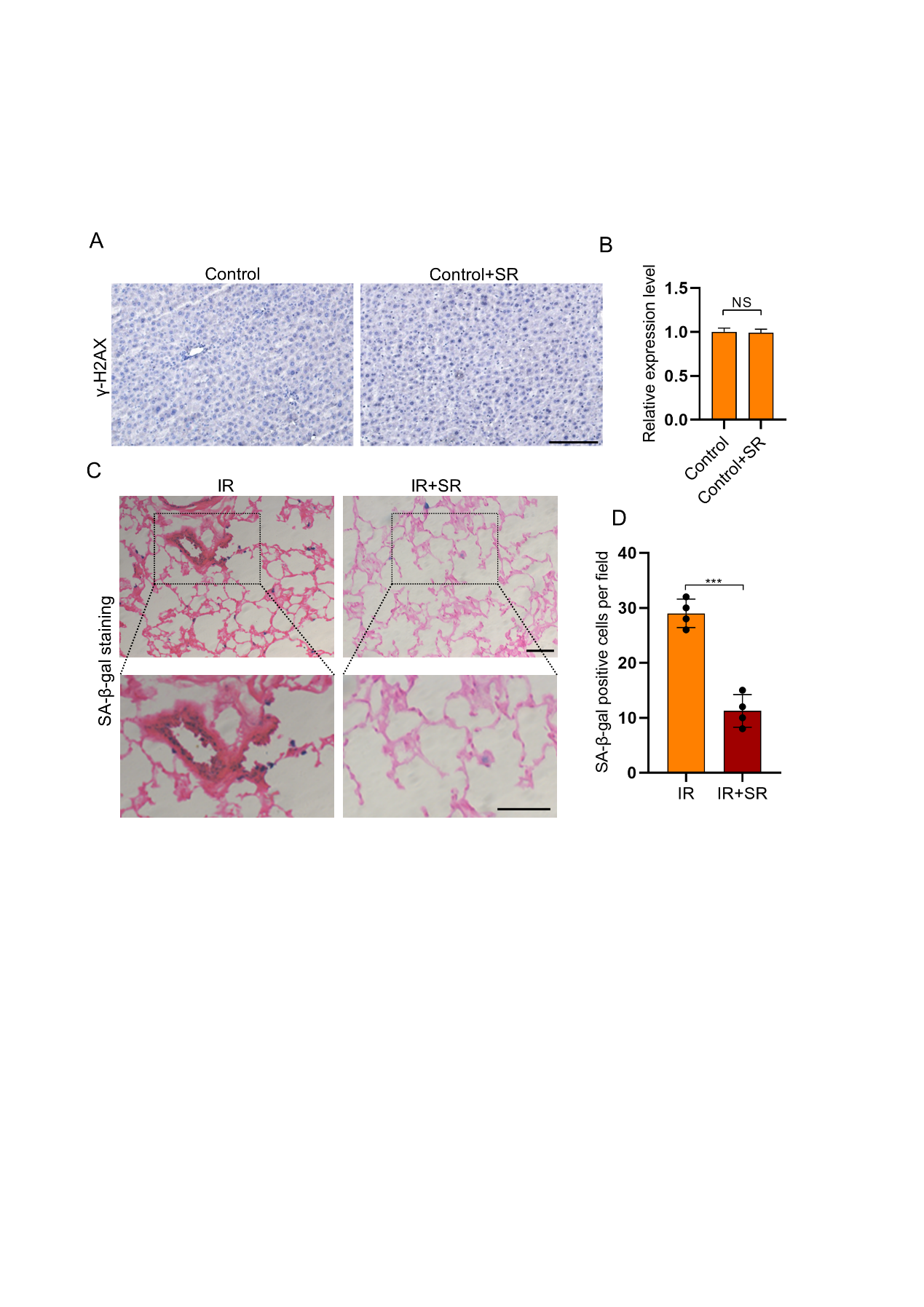


**Supplementary Figure 17. SR9009 suppresses the IR-induced senescence in vivo.** (A) Immunohistochemistry staining of DNA damage factor γ-H2AX in livers of Control and Control+SR9009 group using antibodies shown left. Scale bar, 100 μm. (B) Quantitative analysis of Immunohistochemistry staining. (C) C57BL/6 mice were administered SR9009 every other day. Then induced by IR to promote senescence *in vivo*. Two weeks after ionizing irradiation, the lung was analyzed by SA-β-gal staining. Scale bar, 100 μm. (D) Quantitative analysis of SA-β-Gal positive cells of lung tissues. The results are representative of three independent experiments. *P < 0.05, **P < 0.01, ***P < 0.001, ****P < 0.0001; Student’s t-test.


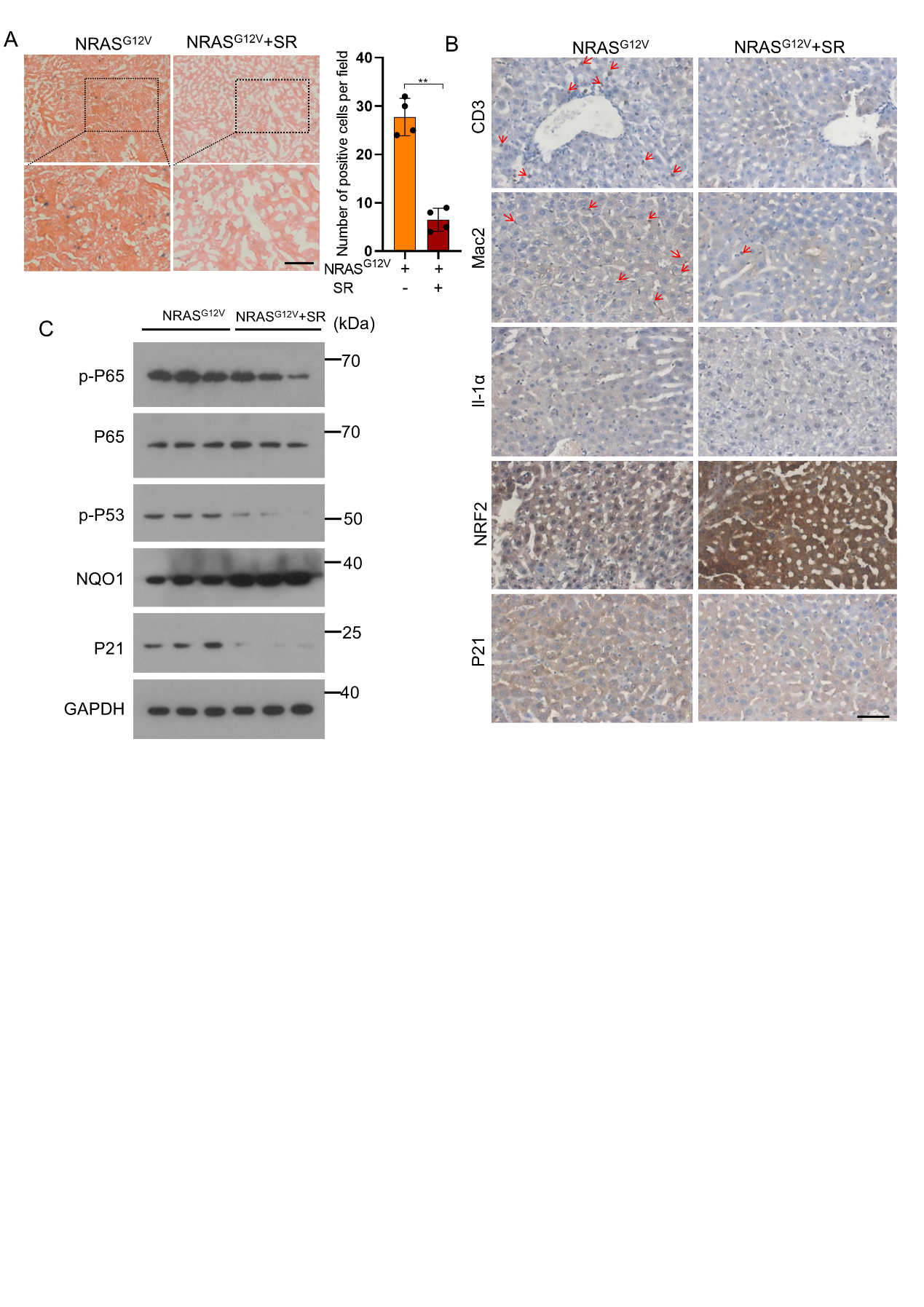


**Supplementary Figure 18. SR9009 suppresses the Nras^G12V^-induced senescence in vivo.** (A) C57BL/6 mice were administered SR9009 every other day. Then induced by the Nras^G12V^ transposon element to promote senescence *in vivo*. One week later, the livers were analyzed by SA-β-gal staining. Scale bar, 50 μm (left). Quantitative analysis of SA-β-Gal positive cells of liver tissues (right). (B) Western blot analysis of proteins indicated on the left. GAPDH was used as loading control. (C) Immunohistochemistry staining of indicated genes in livers using antibodies shown left. Scale bar, 100 μm.


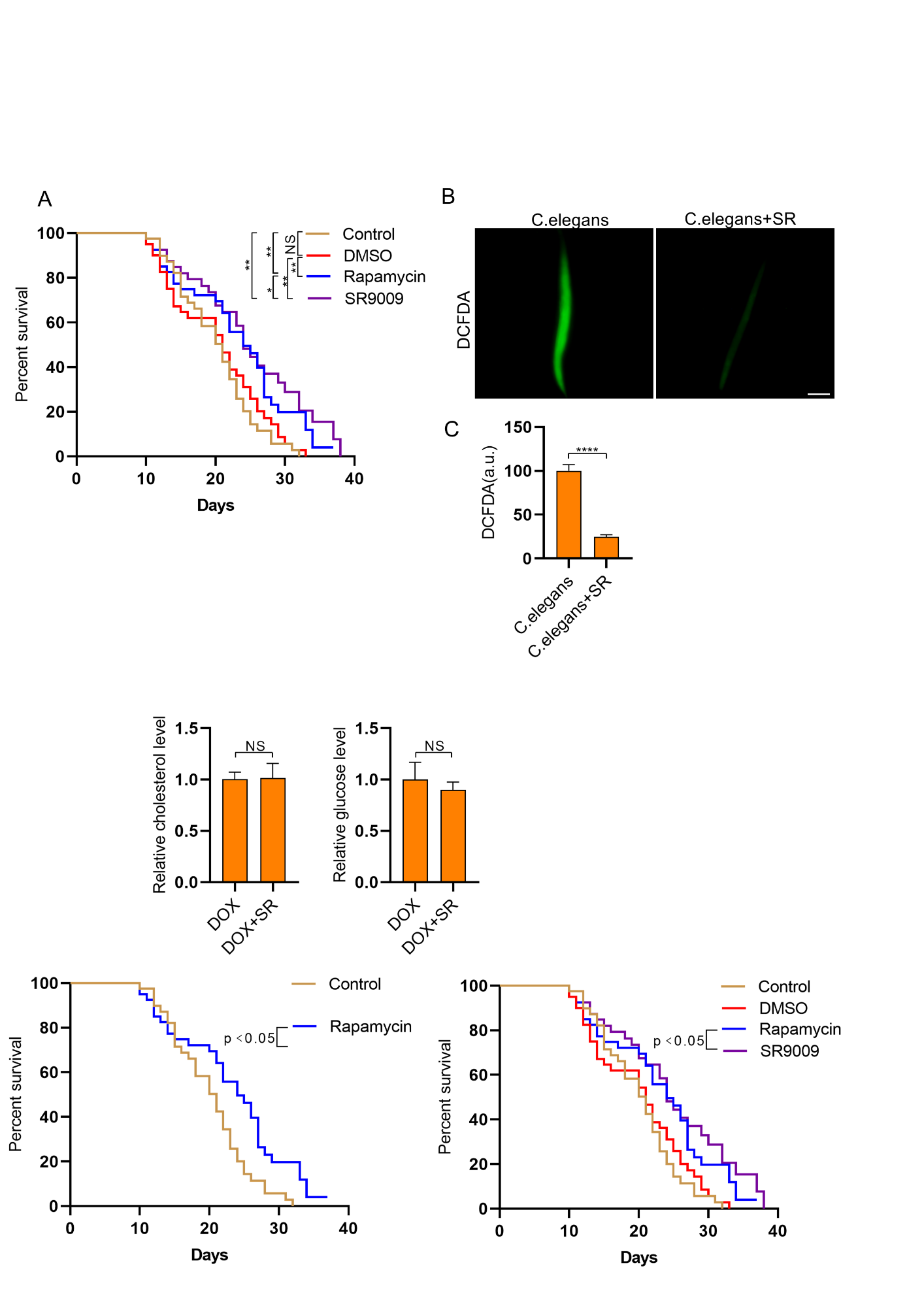


**Supplementary Figure 19. SR9009 extends lifespan of C. elegans**. (A) Survival curves of *C. elegans* treated without or with Rapamycin and SR9009. Data were analyzed using log-rank test. *P < 0.05, **P < 0.01. (B) DCFDA-based ROS quantification of *C. elegans* treated with SR9009 or not. (C) Quantitative analysis of reactive oxygen species (ROS) level. Results are representative of three independent experiments.

| **Gene** | **Forward (5’-3’)** | **Reverse (5’-3’)** |
| --- | --- | --- |
| Human IL-1α | AGATGCCTGAGATACCCAAAACC | CCAAGCACACCCAGTAGTCT |
| Human IL-1β | ATGATGGCTTATTACAGTGGCAA | GTCGGAGATTCGTAGCTGGA |
| Human RPL13A | TCGTACGCTGTGAAGGCATC | CAGCATACCTCGCACGGTC |
| Human ICAM-1 | TGACCGTGAATGTGCTCTCC | TCCCTTTTTGGGCCTGTTGT |
| Human IFN-β | AAACTCATGAGCAGTCTGCA | AGGAGATCTTCAGTTTCGGAGG |
| Human IL-8 | AAGGAAAACTGGGTGCAGAG | ATTGCATCTGGCAACCCTAC |
| Human HRAS | CAGATCAAACGGGTGAAGGAC | GCCTGCCGAGATTCCACAG |
| Human NR1D1 | ATCGTCCGCATCAATCGCAA | CTGCTTCTCTCGTTTGGGGAT |
| Human KLF4 | CGGACATCAACGACGTGAG | GACGCCTTCAGCACGAACT |
| Human C-myc | GGCTCCTGGCAAAAGGTCA | CTGCGTAGTTGTGCTGATGT |
| Human POU5F1 | GGGAGATTGATAACTGGTGTGTT | GTGTATATCCCAGGGTGATCCTC |
| Human SIRT1 | TGTGTCATAGGTTAGGTGGTGA | AGCCAATTCTTTTTGTGTTCGTG |
| Human FASN | AAGGACCTGTCTAGGTTTGATGC | TGGCTTCATAGGTGACTTCCA |
| Human SCD1 | GACGATGAGCTCCTGCTGTT | CTCTGCTACACTTGGGAGCC |
| Human ULK1 | AAGCACGATTTGGAGGTCGC | TGATTTCCTTCCCCAGCAGC |
| Human ULK3 | TGAAGGAGCAGGTCAAGATGA | GCTACGAACAGATTCCGACAG |
| Human BECN1 | CCATGCAGGTGAGCTTCGT | GAATCTGCGAGAGACACCATC |
| Human MMP-1 | AGCCTTCCAACTCTGGAGTAATGT | CCGATGATCTCCCCTGACAA |
| Human MMP-3 | CCCACCTTACATACAGGATTGTGA | CCCAGACTTTCAGAGCTTTCTCA |
| Human CCL-2 | AAGACCATTGTGGCCAAGGA | TTCGGAGTTTGGGTTTGCT |
| Human CXCL-2 | AGAATGGGCAGAAAGCTTGTCT | CCTTCTGGTCAGTTGGATTTGC |
| Human CXCL-1 | AGGGAATTCACCCCAAGAAC | TGGATTTGTCACTGTTCAGCA |
| Human IL-6 | ACTCACCTCTTCAGAACGAATTG | CCATCTTTGGAAGGTTCAGGTTG |
| Human CXCL-3 | CCAAACCGAAGTCATAGCCAC | TGCTCCCCTTGTTCAGTATCT |
| human CXCL10 | CCAGAATCGAAGGCCATCAA | CATTTCCTTGCTAACTGCTTTCAG |
| Human NRF2 | TCCAGTCAGAAACCAGTGGAT | GAATGTCTGCGCCAAAAGCTG |
| Human NQO1 | GAAGAGCACTGATCGTACTGGC | GGATACTGAAAGTTCGCAGGG |
| Human HO-1 | AGTTCAAGCAGCTCTACCGC | GCAACTCCTCAAAGAGCTGGAT |
| Mouse IL-1α | TCTCAGATTCACAACTGTTCGTG | AGAAAATGAGGTCGGTCTCACTA |
| Mouse IL-1β | GAAATGCCACCTTTTGACAGTG | TGGATGCTCTCATCAGGACAG |
| Mouse IL-6 | CTGCAAGAGACTTCCATCCAG | AGTGGTATAGACAGGTCTGTTGG |
| Mouse CXCL-1 | ACTGCACCCAAACCGAAGTC | TGGGGACACCTTTTAGCATCTT |
| Mouse CD45 | CGCGGTGTAAAACTCGTCAA | CCCCAAATCTGTCTGCACATT |
| Mouse CD3 | CAAGAGCTGCCTCAGAAGCA | CGAGAAATCCTGGAGCACCA |

Supplementary Table 1. List of Quantitative Real-Time PCR primers

Supplementary Table 2. Antibodies for western blotting, Immunofluorescence, and Immunohistochemistry

| Western blotting Antibodies | SOURCE | IDENTIFIER |
| --- | --- | --- |
| p-mTOR (Ser2448) (Rabbit) (1:1000) | SAB | Cat #11221 |
| mTOR (Rabbit) (1:1000) | Proteintech | Cat #20657-1-AP |
| p-ATM(Ser1981) (Rabbit) (1:1000) | Epitomics | Cat #EP1890Y |
| ATM (Rabbit) (1:1000) | CST | Cat #2873 |
| p-p53(Ser15) (Rabbit) (1:1000) | CST | Cat #82530 |
| AKT (Rabbit) (1:1000) | CST | Cat #4691 |
| p-AKT (Rabbit) (1:1000) | CST | Cat #4060 |
| P21 (Rabbit) (1:1000) | Proteintech | Cat #10355-1-AP |
| P21 (Rabbit) (1:1000) | Proteintech | Cat #10883-1-AP |
| γ-H2AX (Rabbit) (1:1000) | CST | Cat #9718 |
| H3K9Me3 (Rabbit) (1:1000) | Abclonal | Cat #A2360 |
| P65 (Rabbit) (1:3000) | CST | Cat #10745-1-AP |
| p-P65 (Rabbit) (1:1000) | CST | Cat #3033 |
| NRF2 (Rabbit) (1:1000) | CST | Cat #ab62352 |
| KEAP1 Rabbit (1:1000) | CST | Cat #8047 |
| HRAS (Rabbit) (1:2000) | Proteintech | Cat #18295-1-AP |
| NR1D1 (Rabbit) (1:3000) | Proteintech | Cat #14506-1-AP |
| NR1D2 (Rabbit) (1:3000) | Proteintech | Cat #13906-1-AP |
| P38 (Rabbit) (1:1000) | Proteintech | Cat #14064-1-AP |
| p-P38 (Rabbit) (1:1000) | CST | Cat ##4511 |
| NQO1 (Rabbit) (1:1000) | Proteintech | Cat #11451-1-AP |
| HO-1 (Rabbit) (1:1000) | Proteintech | Cat #10701-1-AP |
| Immunofluorescence or IHC Antibodies SOURCE IDENTIFIER | | |
| γ-H2AX (Rabbit) (1:200) | CST | Cat #9718 |
| Ki67 (Rabbit) (1:200) | Abcam | Cat ab15580 |
| H3K9Me3 (Rabbit) (1:200) | Abclonal | Cat #A2360 |
| NRF2 (Rabbit) (1:200) | Abcam | Cat #ab62352 |
| NQO1 (Rabbit) (1:400) | Proteintech | Cat #11451-1-AP |
| HO-1 (Rabbit) (1:400) | Proteintech | Cat #10701-1-AP |
| Mac2 Rabbit (1:200) | Proteintech | Cat #14979-1-AP |
| CD3 Rabbit (1:200) | Abcam | Cat #ab11089 |
| IL-1α (1:400) | Bioss | Cat #bs-4946R |
| CD3 Rabbit (1:200) | Abcam | Cat #ab11089 |
| IL-1α (1:400) | Bioss | Cat #bs-4946R |
